# Local Packing Density Is the Main Structural Determinant of the Rate of Protein Sequence Evolution at Site Level

**DOI:** 10.1155/2014/572409

**Published:** 2014-07-09

**Authors:** So-Wei Yeh, Tsun-Tsao Huang, Jen-Wei Liu, Sung-Huan Yu, Chien-Hua Shih, Jenn-Kang Hwang, Julian Echave

**Affiliations:** ^1^Institute of Bioinformatics and Systems Biology, National Chiao Tung University, Hsinchu 30050, Taiwan; ^2^Center for Bioinformatics Research, National Chiao Tung University, Hsinchu 30050, Taiwan; ^3^Escuela de Ciencia y Tecnología, Universidad Nacional de San Martín, Martín de Irigoyen 3100, San Martín, 1650 Buenos Aires, Argentina

## Abstract

Functional and biophysical constraints result in site-dependent patterns of protein sequence variability. It is commonly assumed that the key structural determinant of site-specific rates of evolution is the Relative Solvent Accessibility (RSA). However, a recent study found that amino acid substitution rates correlate better with two Local Packing Density (LPD) measures, the Weighted Contact Number (WCN) and the Contact Number (CN), than with RSA. This work aims at a more thorough assessment. To this end, in addition to substitution rates, we considered four other sequence variability scores, four measures of solvent accessibility (SA), and other CN measures. We compared all properties for each protein of a structurally and functionally diverse representative dataset of monomeric enzymes. We show that the best sequence variability measures take into account phylogenetic tree topology. More importantly, we show that both LPD measures (WCN and CN) correlate better than all of the SA measures, regardless of the sequence variability score used. Moreover, the independent contribution of the best LPD measure is approximately four times larger than that of the best SA measure. This study strongly supports the conclusion that a site's packing density rather than its solvent accessibility is the main structural determinant of its rate of evolution.

## 1. Introduction

The evolutionary divergence of protein amino acid sequences is subject to purifying selection against amino acid substitutions imposed by functional and biophysical constraints [1–6]. Due to such constraints, the sites (residues) of a protein amino acid sequence differ in their evolutionary rate (the number of amino acid substitutions per unit of evolutionary time). As a result, multiple alignments of evolutionary related (homologous) proteins show clear site-dependent conservation patterns. Typically, only a few sites are directly related to function and their high conservation is due to direct function-specific selection. Mutations at most other sites affect fitness indirectly through their effect on the protein's folding, stability, structure, or dynamics [6]. Here, we focus on the effect of structural constraints on site-specific sequence divergence.

There are two structural properties that have emerged as the best candidates to account for site-specific rates of evolution: Solvent Accessibility (SA) and Local Packing Density (LPD). Several studies have shown that site-specific substitution rates correlate with SA, measured by the Relative Solvent Accessibility (RSA) [7–11]. Generally, RSA is considered to be the main structural determinant of evolutionary rate at site level. However, site-specific sequence variability has also been reported to correlate significantly with LPD, measured using either the Contact Number (CN) [9, 11, 12] or the Weighted Contact Number (WCN) [11, 13].

Of the cited studies, the only two that compared SA and LPD measures as determinants of site-specific evolutionary rates found opposite results [9, 11]. Franzosa and Xia considered several structural measures regarding their correlation with rates of evolution and found RSA and CN to be the best correlates, with RSA performing slightly better but CN making a significant independent contribution [9]. Moreover, they found that the other structural measures had either no effect or no independent effect on sequence variability. In contrast, Yeh et al. compared RSA with two LPD measures, WCN and CN, on a larger and more divergent dataset of proteins with much better signal and found that both LPD measures correlate better with evolutionary rates than RSA [11]. Moreover, they showed that once LPD is controlled for, the independent contribution of RSA is very small. Furthermore, we recently developed a mechanistic model of protein evolution that explains why rate of evolution is related to LPD [14]. The purpose of the present work is to perform a thorough assessment of the thesis that the packing density of a protein site rather than its solvent accessibility is the main structural determinant of its sequence variability.

There are different ways to quantify sequence variability, SA, and LPD. In a previous study [11], we quantified sequence variability using rates of evolution, RSA values were calculated following Ramsey et al. [10], and we used two LPD measures: WCN and CN with 13 Å cut-off radius. To further assess the packing density versus solvent accessibility as determinants of evolutionary rates issue, here we consider other measures of sequence variability, SA, and LPD. First, we considered five popular measures of sequence variability/conservation that differ methodologically and conceptually [15–17]. Second, we considered four measures of SA: the Absolute Solvent Accessibility (ASA) and the three different measures of RSA [18]. Finally, we considered the effect of changing the cut-off radius used in the definition of CN. Given a sequence or structural measure *X*_*s*_ for each site of a protein's sequence, one can obtain a site-dependent profile *X* = (*X*_1_, *X*_2_,…, *X*_*N*_) for a protein of length *N*. The sequence and structure profiles we consider are summarized in Table 1.

We compared sequence and structural profiles for a diverse representative dataset of monomeric enzymes. For each protein, we compared site-dependent structural profiles with sequence variability profiles, quantified their similarities, and analyzed the resulting data to address the two questions. First, what are the best measures of sequence variability for the sake of studying the sequence-structure evolutionary relationship at site level? Second, what are the structural measures that best quantify the structural evolutionary constraints on sequence divergence? More specifically, does this more thorough analysis support the conclusion that LPD measures outperform SA measures as quantifiers of site-specific evolutionary constraints on sequence divergence?

We found significant sequence-structure correlations regardless of the specific method used to estimate LPD, SA, and sequence variability. Among the sequence variability measures, the ones that take into account the topology of the phylogenetic tree lead to significantly higher sequence-structure correlations with all structural profiles. Regarding structural properties, LPD measures clearly outperform SA measures as predictors of sequence variability, regardless of the specific sequence variability measure used. Therefore, this study provides strong support to our previous finding that site-specific evolutionary rates are determined mainly by packing density rather than solvent accessibility [11].

## 2. Materials and Methods

### 2.1. Dataset

We used the nonredundant dataset of 216 monomeric enzymes of our previous study [11]. The pairwise sequence identity of all pairs of proteins of the dataset is less than 25%. The X-ray structures have less than five missing residues, and they are monomeric (i.e., their biological unit is a single chain). The lengths range from 96 to 1287 sites, with a mean of 361 sites. There are enzymes of all six main EC classes [19] and domains of all main SCOP structural classes [20]. Details of the dataset can be found in Table S1 in Supplementary Material available online at http://dx.doi.org/10.1155/2014/572409. This set is representative of soluble globular monomeric enzymes.

### 2.2. Multiple Sequence Alignment

For each protein of the dataset, a multiple sequence alignment (MSA) was obtained following the ConSurf protocol [21, 22]. First, PSI-BLAST [23] with an *E*-value cut-off of 10^−3^ and three iterations was used to retrieve homologous sequences from the Clean_Uniprot database [24]. Second, all sequences that satisfy the following criteria were removed: (1) sequences with more than 95% identity to the query sequence; (2) sequences shorter than 60% of the query sequence; (3) fragment sequences that overlap by under 10%. Third, CD-HIT [25] was used to select up to maximum 300 most significant representative sequences. Finally, the MSA was obtained using MUSCLE [26].

### 2.3. Sequence Profiles

For each protein, we used its MSA to calculate five sequence variability profiles separately, which are summarized in Table 1 and described here.

*ConSurf*. *CS* = (*CS*_1_, *CS*_2_,…, *CS*_*N*_), where *CS*_*i*_ is the relative rate of evolution of site *i* and *N* is the number of sites. Given the MSA, the site-specific relative rates are calculated using Rate4Site [27, 28]. Rate4Site builds the phylogenetic tree using the neighbor-joining algorithm and estimates the rates using an empirical Bayesian method and the JTT substitution matrix.

*Real-Valued Evolutionary Trace*. *ET* = (*ET*_1_, *ET*_2_,…, *ET*_*N*_) is a profile of scores that measures the variability of each site taking into account the topology of the phylogenetic tree and the variability within groups of sequences defined by such topology [29]. Given the MSA, the tree's topology is obtained using the UPGMA algorithm [30]. The tree is used to define groups of sequences, the Shannon entropy is used to measure within-group variability, and ET is a sum of such entropies with group-dependent weights. Branch-lengths are not taken into account in the calculation.

*Karlin & Brocchieri Sum-of-Pairs*. *KBSP* = (*KBSP*_1_, *KBSP*_2_,…, *KBSP*_*N*_) consists of conservation scores obtained by adding the amino acid similarity scores from a normalized substitution matrix over all pairs of sequences of the MSA [31]. In this study, we used the JTT250 substitution matrix [16].

*Valdar & Thornton Sum-of-Pairs*. *VTSP* = (*VTSP*_1_, *VTSP*_2_,…, *VTSP*_*N*_) consists of conservation scores obtained by adding over sequence pairs the amino acid similarity scores from a normalized substitution matrix [32]. The difference between the VTSP and the KBSP is that VTSP uses a different procedure to normalize the substitution matrix and weights sequences to reduce possible biases introduced by closely related sequences. We used the JTT250 substitution matrix [16].

*Entropy*. *EN* = (*EN*_1_, *EN*_2_,…, *EN*_*N*_) consists of variability scores measured by Shannon's information entropy obtained from the site-specific amino acid frequencies [33]. The entropy at each sequence position is defined as *EN*_*i*_ = −∑_*a*_ *f*_*ia*_ln⁡*f*_*ia*_, where *f*_*ia*_ is the frequency of an amino acid type *a* at sequence position *i*. The entropy is zero for a completely conserved site and increases with variability.

### 2.4. Structural Profiles

For each protein, we used its PDB file [34] to calculate the structural profiles summarized in Table 1 and described here.

#### 2.4.1. Local Packing Density

*Weighted Contact Number*. *WCN* = (*WCN*_1_, *WCN*_2_,…, *WCN*_*N*_) is a local packing density profile defined in [35]. WCN of residue *i* is *WCN*_*i*_ = ∑_*j*≠*i*_^*N*^1/*r*_*ij*_^2^, where *r*_*ij*_ is the distance between the *C*_*α*_ of residues *i* and *j* and *N* is the number of residues.

*Contact Number*. *CN* = (*CN*_1_, *CN*_2_,…, *CN*_*N*_) is a local packing density profile. The CN of a site is defined as the number *C*_*α*_ within a spherical neighbourhood of cut-off radius *r*_0_. We calculated CN values with *r*_0_ ranging from 9 to 30 Å, with an interval of 1 Å to find the optimum cut-off radius.

#### 2.4.2. Solvent Accessibility

*Absolute Solvent Accessibility*. *ASA* = (*ASA*_1_, *ASA*_2_,…, *ASA*_*N*_) is a solvent accessibility profile. The absolute solvent accessibility of a site is computed by rolling a 1.4 Å sphere, simulating a water molecule, over the residue's molecular surface. We used the program DSSP [36].

*Relative Solvent Accessibility Profile*. *RSA* = (*RSA*_1_, *RSA*_2_,…, *RSA*_*N*_) consists of site-specific measures of solvent accessibility. The Relative Solvent Accessibility (RSA) of a residue is its ASA divided by the maximum ASA for the given amino acid type. We used three different values of the maximum ASA: those of Rose et al. [37], Miller et al. [38], and Tien et al. [18], leading to, respectively, three different RSA profiles: RSA^R^, RSA^M^, and RSA^T^.

### 2.5. Profile Comparison

For each protein, we compared all sequence profiles with all structural profiles. To reduce noise, all profiles were smoothed using a sliding window of size three as recommended in Pei and Grishin [15]. The similarity between two profiles was quantified using Pearson's correlation coefficient, which ranges from −1 for perfectly anticorrelated profiles to 1 for perfectly correlated ones. For unrelated profiles, the expected value is 0. Since KBSP and VTSP are measures of conservation rather than variability, we changed their sign, so that the most conserved sites have lower score. We did the same for WCN and CN so that the sites with higher local packing density, which are expected to be more conserved, get the lowest score. In this way, all significant relationships will result in positive correlations.

Pearson correlations are especially useful for linear relationships between the variables compared. In the present case, their use is justified because site-specific rates of evolution are linearly related to both RSA and LPD [9, 10, 14]. However, in order to further support the conclusions regardless of whether the relationship is linear or not, we also calculated the rank-based Spearman correlation coefficients between the different profiles and performed nonparametric rank-based statistical assessments described next.

### 2.6. Statistical Assessment to Compare Two Predictor Variables

Given a reference sequence (structure) profile *y*, we compared two structural (sequence) profiles *x*_1_ and *x*_2_ using their Pearson or Spearman correlation coefficients *ρ*(*y*, *x*_1_) and *ρ*(*y*, *x*_2_). To assess whether *ρ*(*y*, *x*_1_) > *ρ*(*y*, *x*_2_), we performed three statistical tests. First, we used a paired *t*-test to assess whether the means over proteins satisfy 〈*ρ*(*y*, *x*_1_)〉 > 〈*ρ*(*y*, *x*_2_)〉. Second, we calculated the proportion proteins for which *ρ*(*y*, *x*_1_) > *ρ*(*y*, *x*_2_) and used a binomial test to assess whether such proportion is larger than 50%. Third, we tested whether *ρ*(*y*, *x*_1_) > *ρ*(*y*, *x*_2_) using Wilcoxon's signed-rank test with matched pairs. Wherever we use the term “significant” we mean that the *P* value of the test that gives the worst *P* value is smaller than 0.01.

We note here that the *P* value of the *t*-test is strictly valid only under the assumption of a normal distribution of the random variable considered, which in the present case is a good approximation. Despite this, we note that in general the *t*-test is robust with respect to departures from normality. Therefore the *t*-test is suitable for the present case. In spite of this, for the sake of a more thorough assessment, we also used the binomial test and Wilcoxon's test that do not depend on any assumptions about the form of the underlying distributions.

### 2.7. Statistical Assessment of the Redundant and Independent Contributions of LPD and RSA to Rates of Evolution

To address the issue of the relative importance of WCN (the best LPD measure) and RSA^T^ (the best RSA measure) as predictors of CS (the best sequence variability measure), we used a variance partitioning analysis in which the overall explained variance is split into overlapping and unique contributions of WCN and RSA^T^. For the case of a linear fit CS ~ WCN + RSA^T^, the variance of CS explained together by WCN and RSA^T^ is the square of the bivariate correlation coefficient *R* ^2^. This can be partitioned into the sum of three contributions:

(1) R2=ρ2(CS,WCN  or  RSAT)+ρ2(CS,WCN ∣ RSAT)+ρ2(CS,RSAT ∣ WCN).

The first term accounts for the redundant contribution of the independent variables and is due to the fact that they correlate with each other. The last two terms are the square semipartial correlations of CS with each of the independent variables controlling the other and they represent their unique contributions [11, 39, 40]. Another way to interpret this partitioning is that the unique contribution of a variable is the increase in *R* ^2^ that results from adding that variable to the linear fit.

## 3. Results and Discussion

For each of the 216 proteins of our dataset, we obtained the structure from the Protein Data Bank [34] and built a multiple sequence alignment. Given a protein, we calculated all the site-dependent sequence and structural profiles summarized in Table 1 and described in Section 2. The sequence variability profiles are the ConSurf rate of evolution (CS), the Evolutionary Trace score (ET), the Karlin & Brocchieri Sum-of-Pairs score (KBSP), the Valdar & Thornton Sum-of-Pairs score (VTSP), and the Shannon Entropy (EN). The structural profiles are the Weighted Contact Number (WCN), the simpler Contact Number (CN) with varying cut-off radii, the Absolute Solvent Accessibility (ASA), and three measures of Relative Solvent Accessibility: RSA^R^, RSA^M^, and RSA^T^.

For each protein, we calculated Pearson's correlation coefficients between each sequence profile and each structural profile.  Table 2 shows the average over proteins of such correlations. Similar results are found using Spearman correlations (Table S8). All values are significantly positive. Therefore, in general, all structural profiles are significantly correlated with all sequence profiles. However, there are significantly different sequence-structure correlations depending on the sequence and structural measures compared.

### 3.1. Comparison of Sequence Measures

**Figure 1:**
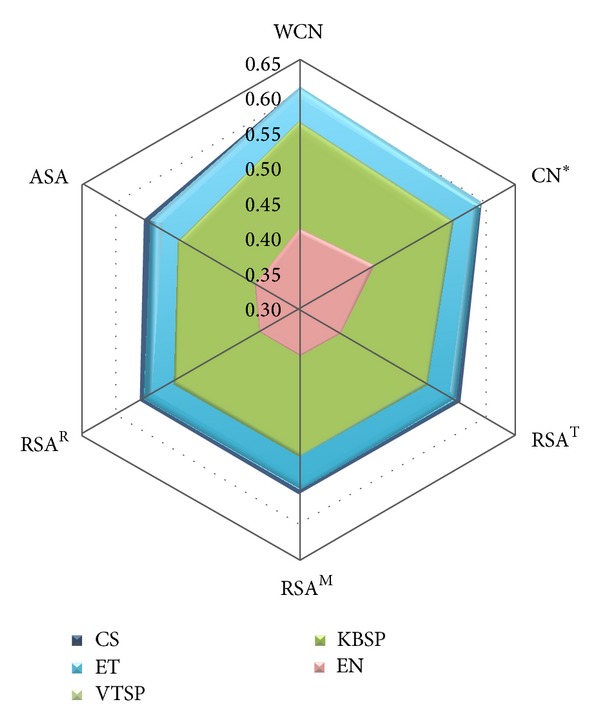
Comparison of sequence variability profiles by their average Pearson's correlation coefficients with different structural profiles. The sequence variability scores (listed in the figure legend) are ConSurf rate of evolution (CS), Evolutionary Trace score (ET), Karlin & Brocchieri Sum-of-Pairs score (KBSP), Valdar & Thornton Sum-of-Pairs score (VTSP), and Entropy (EN). The structural properties (the apices of the hexagon) are Weighted Contact Number (WCN), Contact Number (CN), Relative Solvent Accessibility (RSA), and Absolute Solvent Accessibility (ASA). The asterisk mark on CN means that the cut-off radius was chosen to maximize each CN-sequence average correlation. The cut-off radii for CS, ET, KBSP, VTSP, and EN are 19 Å, 19 Å, 18 Å, 18 Å, and 20 Å, respectively. Superscript letters distinguish RSA profiles obtained using different methods.

What are the sequence variability measures resulting in higher sequence-structure correlations? Table 2 (reading it rowwise) and Figure 1 show the effect of different sequence measures on average sequence-structure correlations. The five sequence profiles cluster into three groups. For all structural measures, Shannon's entropy EN gives by far the worst sequence-structure correlations. The Sum-of-Pairs scores KBSP and VTSP give almost identical sequence-structure correlations, much better than EN. CS and ET lead to the highest sequence-structure correlations, with CS either similar to or slightly better than ET depending on structural measure. As we will see in the next section, the best solvent accessibility measure is RSA^T^ and the best packing density measure is WCN. Regarding the correlation with RSA^T^, the means follow the order CS≅ET > KBSP≅VTSP > EN, as can be seen from Table 2. This order is supported by all statistical tests (Table S2). Further, a protein-by-protein comparison shows that CS and ET have higher correlations with RSA^T^ than KBSP and VTSP for more than 70% of the proteins and the latter are better than EN for more than 80% of the cases, both values being significantly larger than 50% according to a binomial test (Table S2). A similar assessment shows that, with respect to their correlations with WCN, sequence profiles, again, follow the order CS≅ET > KBSP≅VTSP > EN, as seen in Table 2, and supported by all statistical tests (Table S3). The same conclusions are reached using Spearman coefficients (see Figure S1, Table S8, Table S9, and Table S10).

To interpret the previous results, we notice that CS and ET are the only methods that take into account the topology of the phylogenetic tree, which seems to be the key factor responsible for the improvement over the Sum-of-Pairs methods KBSP and VTSP. Entropy, EN, which takes into account neither the tree topology nor the substitution probabilities, gives very poor sequence-structure correlations. Even though difference between CS and ET is in general not statistically significant, CS does give slightly better results (see Figure 1 and Figure S1). Moreover, while ET is an empirical score, CS has a clear evolutionary meaning, since it is the site-specific rate of evolution inferred using a robust Bayesian approach and an explicit evolutionary model. Therefore, we consider CS to be the best measure of sequence variability for the purpose of studying the sequence-structure evolutionary relationship.

### 3.2. Comparison of Structural Measures

**Figure 2:**
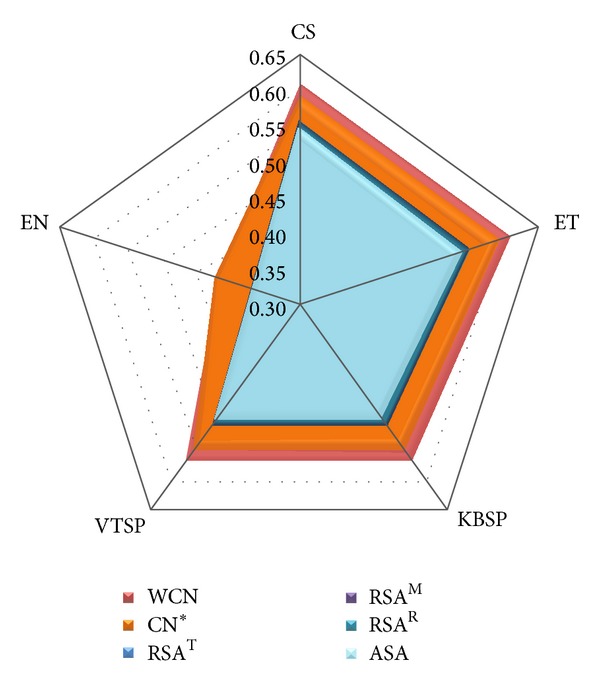
Comparison of structural profiles by their average Pearson's correlation coefficients with different sequence variability profiles. The sequence variability measures (the axes of the pentagon) are ConSurf rate of evolution (CS), Evolutionary Trace score (ET), Karlin & Brocchieri Sum-of-Pairs score (KBSP), Valdar & Thornton Sum-of-Pairs score (VTSP), and Entropy (EN). The structural properties (listed in the figure legend) are Weighted Contact Number (WCN), Contact Number (CN), Relative Solvent Accessibility (RSA), and Absolute Solvent Accessibility (ASA). The asterisk mark on CN means that the cut-off radius was chosen to maximize each CN-sequence average correlation. The cut-off radii for CS, ET, KBSP, VTSP, and EN are 19 Å, 19 Å, 18 Å, 18 Å, and 20 Å, respectively. Superscript letters distinguish RSA profiles obtained using different methods.

What are the structural properties correlating better with sequence variability measures? Inspection of Table 2 (columnwise) and Figure 2 shows clearly that LPD measures (WCN and CN*) correlate, on average, better with all sequence variability measures than SA measures (ASA, RSA^R^, RSA^M^, and RSA^T^). Similar results are seen using Spearman correlations (Table S8 and Figure S2). In the following sections, we perform a more detailed comparison.

#### 3.2.1. Comparison of Solvent Accessibility (SA) Measures

What is the best solvent accessibility measure? From Table 2 and Figure 2, it is clear that all SA measures give similar sequence-structure correlations for all sequence variability measures. Except for EN, which is a poor sequence variability score, RSA measures are larger than ASA (see Table 2), which is supported by all statistical tests (Table S4). Among the relative SA measures, RSA^R^, RSA^M^, and RSA^T^, differences are very small. However, all statistical tests indicate that the best RSA-sequence correlation is obtained using RSA^T^, based on the recent maximum allowed ASA proposed by Tien et al. [18] (Table S4). Thus, regarding sequence-structure correlations, RSA^T^ is the best measure of solvent accessibility. The same conclusions follow from similar analyses of Spearman correlations (Table S8, Figure S2, and Table S11).

#### 3.2.2. Comparison of Local Packing Density (LPD) Measures

What is the best LPD measure? In Table 2 and Figure 2, we show the mean sequence-structure correlations for WCN and CN* with the different sequence variability measures. WCN is a parameter-free measure. CN, on the other hand, depends on a cut-off radius. CN* was obtained by varying the cut-off radius and finding the maximum (Table S5).  Table 2 and Figure 2 show that, except for the poorest sequence variability score EN, WCN correlates, on average, better than CN* for all other site-dependent sequence profiles (Table S6). Moreover, for more than 50% of the proteins, WCN outperforms CN* for all sequence variability measures except EN (Table S6). Similar conclusions follow from analyses of Spearman correlations (Figures S2, Table S8, Table S12, and Table S13). To summarize, WCN is a better LPD measure than CN to study the LPD-sequence relationship.

#### 3.2.3. Weighted Contact Number (WCN) versus Relative Solvent Accessibility (RSA)

To complete this study, we perform a more detailed comparison between the best LPD measure (WCN) and the best SA measure (RSA^T^).  Table 2, Table 3, and Figure 2 clearly show that the mean WCN-sequence correlation coefficients are larger than mean RSA^T^-sequence correlations for all measures of sequence variability. This is supported by all statistical tests (Table S7). Similar conclusions are reached from analyses based on Spearman correlations (Table S8, Figure S2, and Table S14).

**Figure 3:**
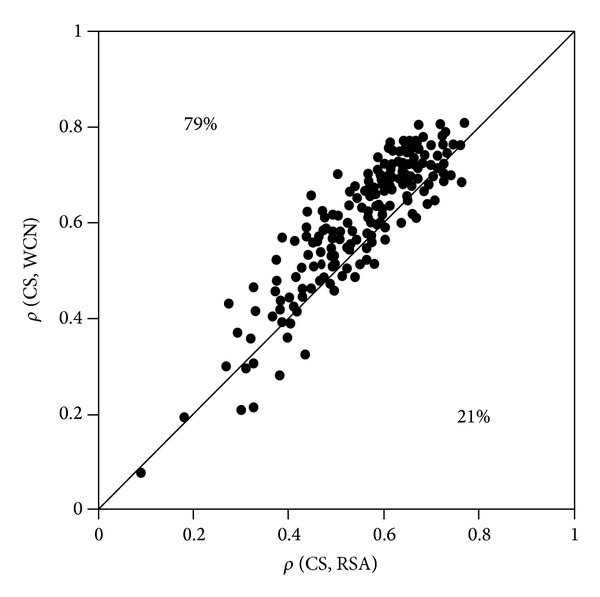
Local packing density versus solvent accessibility as determinants of site-specific evolutionary rates. Points above (below) the diagonal are proteins for which WCN (RSA^T^) correlates better than RSA^T^ (WCN) with the site-specific rates of amino acid substitution as estimated using the phylogenetic-based approach ConSurf (CS). The percentages of points above and below the diagonals are shown.

Since CS is the best sequence variability measure, we compared WCN-CS and RSA^T^-CS correlations protein-by-protein. Results are shown in Figure 3. Counting the number of cases above and below the diagonal, we found that *ρ*(CS, WCN) > *ρ*(CS, RSA^T^) for 171/216 = 79% of cases, and Table 3 indicates that the proportion is significantly larger than 50% (supported by a binomial test, Table S7). The mean sequence-structure correlations are 〈*ρ*(CS, WCN)〉 = 0.61 and 〈*ρ*(CS, RSA) = 0.56. Therefore, both the number of cases and the mean values support that WCN correlates better with site-specific evolutionary rates than RSA. This is further supported by analysis based on Spearman correlations (Figure S3, Table S14).

#### 3.2.4. Joint and Unique Contributions of WCN and RSA

Despite LPD measures being better than SA measures, it is possible that both contribute significantly as determinants of the rate of evolution. Therefore, to finish, we consider the extent to which WCN and RSA^T^ provide overlapping and independent contributions to the explained variance of CS. For this purpose, we performed a variance partitioning analysis based on semipartial correlations (see Section 2). In a previous study, we used such analysis to compare WCN and RSA^M^ [11]. Here, we showed that the best SA measure is RSA^T^, so that it is necessary to repeat the analysis. Results are shown in Table 4. The total explained variance is *R* ^2^ = 0.408. As a result of the large WCN-RSA^T^ correlations, the redundancy term is the largest. WCN accounts uniquely for 19.5% of the explained variance, while RSA's unique contribution is 5.3%. Therefore, the unique contribution of the best LPD measure, WCN, is almost four times larger than the unique contribution of the best SA measure, RSA^T^. Another way to interpret these results is that going from a one-variable CS ~ WCN linear fit to a two-variable CS ~ WCN + RSA^T^ fit increases the explained variance only by 5.3% (from *R* ^2^ = 0.392 to *R* ^2^ = 0.408) at the cost of introducing an extra parameter and possibly overfitting. Similar results are obtained using Spearman correlations (Table S15).

## 4. Conclusion

Franzosa and Xia studied many structural measures that characterize the microenvironment of protein sites looking for the main structural determinants of evolutionary rate at site level [9]. They found that the only two structural properties with significant independent contributions are RSA, a measure of solvent accessibility, and CN (with a 13 Å cut-off radius), a measure of packing density. They concluded that, in agreement with the well-known observation that surface sites evolve more rapidly than buried ones, the main determinant is RSA, with CN having a smaller but significant independent contribution. In contrast, in recent study, Yeh et al. found that site-specific amino acid substitution rates correlate better with two LPD measures, WCN and CN, than with RSA, suggesting that packing density rather than solvent accessibility would be the main structural constraint [11]. Taking into account the conflicting conclusions of these two studies and considering that there are different ways of scoring sequence variability, packing density, and solvent accessibility, here we performed a more thorough assessment. To this end, we considered five different measures of sequence variability and four measures of solvent accessibility and varied the cut-off radius used to calculate CN. We performed a protein-by-protein comparison of these properties on a representative dataset of 216 structurally and functionally diverse monomeric globular enzymes.

There are several ways to quantify sequence variability. We compared five sequence variability profiles, CS, ET, KBSP, VTSP, and EN, with four solvent accessibility profiles and two local packing density profiles. We found that CS and ET profiles correlate with all structural profiles better than Sum-of-Pairs similarity scores (KBSP and VTSP), which in turn outperform the simple entropy conservation score (EN). The key factor that differentiates CS and ET from the other methods is that they take into account the topology of the phylogenetic tree. Since CS gives slightly better results and, moreover, has a clear evolutionary interpretation—it is the profile of site-specific evolutionary rates—we think that it should be the method of choice, at least for the purpose of investigating the evolutionary sequence-structure relationship.

The main finding of the present work is that LPD measures (WCN and CN) clearly outperform all of the SA measures (ASA, RSA^R^, RSA^M^, and RSA^T^) for all five of the sequence variability measures. Moreover, WCN is the best LPD measure and RSA^T^ the best SA measure. A variance partitioning analysis based on a bivariate fit of evolutionary rate (CS) as a function of both variables shows that WCN has an independent contribution four times larger than RSA. Therefore, the present assessment provides very strong support for the conclusion of Yeh et al. that the main structural determinant of sequence variability is packing density rather than solvent accessibility [11].

From a fundamental point of view, LPD and RSA suggest different mechanisms for the link between structural constraints and sequence variability. RSA is related to overall protein stability, which would suggest a connection between the effect of a mutation and global stability. On the other hand, LPD is related to the interaction energy of a protein site with its local environment [41]. We have recently developed a mechanistic model of evolution that shows that LPD is directly proportional to the mutational stress introduced by a mutation on the protein's active structure. This model provides an explanation for the sequence-LPD link and predicts a linear relationship [14]. Therefore, the findings of the present thorough analysis further support such mechanistic model, which may provide a breakthrough in our understanding of the biophysical mechanism by which protein structure constrains sequence divergence.

In addition to fundamental issues, the present conclusions could be applied to the development of better structure-based models of sequence evolution. For example, Scherrer et al. have developed sequence evolution models that take into account the site-specific RSA values [42]. The present work suggests the development of similar models based on WCN. It would be interesting to see whether WCN-based evolutionary models outperform RSA-based ones. A secondary issue to note in this respect is that, in contrast with RSA, WCN is easier to calculate, since it depends just on the alpha-carbon coordinates, in contrast with RSA, which considers all of the protein's atoms. However, a very recent study shows that it might be possible to obtain RSA measures from coarse-grained representations, which would tackle this computational-cost problem [43].

The fact that using a weighted contact number, WCN, improves over the simpler CN measure of local packing density immediately suggests that other weighting schemes may further improve the structure-sequence correlations. WCN uses 1/*d*_*ij*_^2^ weights. The first obvious generalization is to use other powers, 1/*d*_*ij*_^*n*^; we have tried this and it turns out that *n* = 2 results in the best sequence-structure correlations (unpublished results). Another choice would be to use decaying exponential weights, *e* ^−*ad*_*ij*_^; this does not improve the sequence-structure agreement either (unpublished results). A third possibility would be to use statistical potentials as weights. We used them in the past in a structure-based model of evolution that predicts successfully the site-specific patterns of amino acid replacement but fails to account for the evolutionary rate variation among sites [44–46]. The reason why an inverse squared distance weighting of contacts leads to the LPD measure that best correlates with site-specific rate of evolution is not clear yet and requires further research. Another issue, suggested by one of the reviewers, is the inclusion of correlations of pairs or higher groups of atoms; we think this is a good idea that might deserve further investigation. To finish this paragraph, we mention that a strategy that does significantly improve over WCN as calculated here is to use a two-nodes-per-site representation including for each site its *C*_*α*_ and a second node representing the side chain located either at the *C*_*β*_ or at the center-of-mass of the side chain and calculating WCN for the node representing the side chain rather than *C*_*α*_. This makes sense, since it is the side chain and not the backbone atom which is mutated. Another approach which leads to similar results is to use an anisotropic weighting function that takes into account not only the distance between the reference site and its neighbors but also its relative orientation with respect to a unit vector directed from the site's *C*_*α*_ to its *C*_*β*_ or side-chain center of mass. These results go beyond the scope of the present work and will be published elsewhere.

To finish, we discuss the scope of the present conclusions. We have used only monomeric enzymes. The set is representative of the whole set of monomeric enzymes of known structures, since, starting from this set, we picked them randomly with the only condition of filtering out enzymes with more than 25% sequence identity to avoid redundancies. Above that sequence-identity threshold, proteins are expected to have essentially the same structures, so that there would be no further gain in including them. The protocol used to build this set guarantees that it is representative of the whole set of monomeric enzymes, in the sense of including the different functional and structural classes in the same proportion as in the whole set. This, together with the fact that the uncertainty of a statistical analysis depends on the size of the sample but has no relationship to the size of the population from which the sample was drawn, means that the present conclusions are expected to hold for monomeric enzymes in general and probably for globular monomeric proteins that are not enzymes but have a similar organization, for example, myoglobin, which is monomeric and globular and has an active site. Using monomeric globular proteins, we avoided constraints due to interaction between subunits in multimeric proteins. For the latter, coupling between subunits may affect the correlation between LPD and sequence variability [47]. Thus, an extension of the present study to multimeric proteins might be useful to gain insight into the coevolution between protein subunits. Further research would also be needed for monomeric proteins whose function is related to protein-protein or protein-nucleic acid interactions, which may impose additional constraints.

## Supplementary Material

The supplementary material contains a list of the 216 monomeric enzymes used in the manuscript shown in Table S1. All details of the statistical tests used in this study are shown in Table S2 to S7. The results based on Spearman correlations are shown in Figure S1, S2, and S3, and Table S8 to S15.

## Figures and Tables

**Table 1:** Site-specific properties.

Symbol	Property measured	Name and description
CS	Rate of evolution	ConSurf rate of evolution: estimated rate relative to the overall average, computed using an empirical Bayesian approach using the phylogenetic tree topology and branch lengths and the JTT probability matrix of amino acid substitutions as implemented in the ConSurf web server.
ET	Sequence variability	Real-valued evolutionary trace: sequence variability score computed using a weighted average of sequence entropy with weights accounting for the topology of the phylogenetic tree.
KBSP	Sequence conservation	Karlin & Brocchieri Sum-of-Pairs: sequence conservation score computed by summing amino acid similarity scores over all amino acid pairs of the site's column in a multiple sequence alignment. Similarity scores are obtained using a normalized JTT250 matrix.
VTSP	Sequence conservation	Valdar & Thornton Sum-of-Pairs: sequence conservation score computed by summing amino acid similarity scores over all amino acid pairs of the site's column in a multiple sequence alignment. Sequences are weighted, and similarity scores are obtained using a min-max normalized JTT250 matrix.
EN	Sequence variability	Entropy: Shannon information entropy computed using the amino acid frequencies observed at the site's MSA column.

CN	Local packing	Contact number: the number of *C*_*α*_ within various distances of the site's *C*_*α*_. The cut-off distance ranges from 9 to 30 Å.
WCN	Local packing	Weighted contact number: measure of contact density obtained by summing the inverse square distances between the site's *C*_*α*_ and the rest of the sites of the protein.

ASA	Solvent accessibility	Accessible surface area: solvent accessibility of the site computed by rolling a 1.4-Å sphere over the residue's molecular surface.
RSA	Solvent accessibility	Relative solvent accessibility: solvent accessibility of the site computed by rolling a 1.4-Å sphere over the residue's molecular surface, divided by the maximum value for residues of the same type. We consider three different tables of values of maximum ASA resulting in three RSA measures: RSA^R^, RSA^M^, and RSA^T^.

**Table 2:** Mean structure-sequence Pearson correlations.

Property	Profile	CS	ET	KBSP	VTSP	EN
LPD	WCN	0.608	0.609	0.567	0.567	0.413
	CN∗	0.596	0.596	0.551	0.551	0.422

SA	RSA^T^	0.559	0.553	0.508	0.507	0.365
	RSA^M^	0.558	0.551	0.505	0.504	0.364
	RSA^R^	0.557	0.551	0.505	0.504	0.364
	ASA	0.551	0.542	0.497	0.496	0.371

*For each sequence variability profile, the cut-off radius of CN was chosen to maximize the CN-sequence average correlation coefficients. The cut-off radii for CS, ET, KBSP, VTSP, and EN are 19 Å, 19 Å, 18 Å, 18 Å, and 20 Å, respectively. Values are the structure-sequence Pearson correlation coefficients averaged over all proteins of the dataset.

**Table 3:** Comparison between WCN and RSA^T^ using sequence profiles as reference.

Reference	WCN	RSA^T^	Δ^1^	%^2^
CS	0.608	0.559	0.049∗	79^†^
ET	0.609	0.553	0.056∗	80^†^
KBSP	0.567	0.508	0.059∗	82^†^
VTSP	0.567	0.507	0.060∗	83^†^
EN	0.413	0.365	0.048∗	77^†^

^1^Difference between the mean correlations of WCN and RSA^T^.

^2^The percentage of cases for WCN > RSA^T^.

*p value ≪ 10^−3^ according to a paired *t*-test.

^†^ *P* value ≪ 10^−3^ according to a binomial test.

**Table 4:** Variance partitioning.

Fit	Contribution	*R* ^2^	%
CS~WCN + RSA^T^	Total	0.408 ± 0.010	100
	Common	0.314 ± 0.009	75.20 ± 1.22
	Unique WCN	0.078 ± 0.004	19.49 ± 1.03
	Unique RSA^T^	0.016 ± 0.001	5.31 ± 0.66

NOTE: fit is the bivariate linear fit considered, *R* ^2^ is the explained variance averaged over the dataset of 216 enzymes ± its standard deviation, and % is the proportion of explained variance accounted for by the given contribution.
