# Supplementary material for: Local Packing Density Is the Main Structural Determinant of the Rate of Protein Sequence Evolution at Site Level
Source: Biomed Res Int. 2014 Jul 9;2014:572409. doi: 10.1155/2014/572409 (PMC4119917; doi:10.1155/2014/572409)
Supplement: Supplementary file 1 — The supplementary material contains a list of the 216 monomeric enzymes used in the manuscript shown in Table S1. All details of the statistical tests used in this study are shown in Table S2 to S7. The results based on Spearman correlations are shown in Figure S1, S2, and S3, and Table S8 to S15. [file 572409.f1.pdf]

**TABLE S1**
**A list of the 216 proteins**

| PDB | EC Class | SCOP | Chain | Nsites[1] | Year | Resolution (Å) | R.value | Nseq[2] |
|---|---|---|---|---|---|---|---|---|
| 132L | 3.02.01.0017 | a+b | A | 124 | 1993 | 1.8 | 0.173 | 300 |
| 13PK | 2.07.02.0003 | a/b | A | 415 | 1996 | 2.5 | 0.221 | 300 |
| 1A26 | 2.04.02.0030 | a, a+b | A | 351 | 1998 | 2.25 | 0.168 | 300 |
| 1A2T | 3.01.31.0001 | b | A | 134 | 1998 | 1.96 | 0.169 | 300 |
| 1A4L | 5.99.01.0002 | a/b | A | 349 | 1998 | 2.6 | 0.185 | 300 |
| 1A65 | 1.10.03.0002 | b, b, b | A | 504 | 1998 | 2.23 | 0.16 | 300 |
| 1A8H | 6.01.01.0010 | a, a/b | A | 500 | 1998 | 2 | 0.205 | 300 |
| 1A8S | 1.11.01.0010 | a/b | A | 273 | 1998 | 1.8 | 0.173 | 300 |
| 1AF7 | 2.01.01.0080 | a, a/b | A | 274 | 1997 | 2 | 0.2 | 300 |
| 1AGM | 3.02.01.0003 | a | A | 470 | 1994 | 2.3 | 0.124 | 300 |
| 1AGY | 3.01.01.0000 | a/b | A | 197 | 1997 | 1.15 | 0.175 | 300 |
| 1AH7 | 3.01.04.0003 | a | A | 245 | 1997 | 1.5 | 0.203 | 83 |
| 1AJ0 | 2.05.01.0015 | a/b | A | 282 | 1997 | 2 | 0.206 | 300 |
| 1AK0 | 3.01.30.0001 | a | A | 264 | 1997 | 1.8 | 0.207 | 300 |
| 1AKO | 3.01.11.0002 | a+b | A | 268 | 1997 | 1.7 | 0.169 | 300 |
| 1AMY | 3.02.01.0001 | b, a/b | A | 403 | 1994 | 2.8 | 0.153 | 300 |
| 1AQ2 | 4.01.01.0049 | a/b, a/b | A | 534 | 1997 | 1.9 | 0.218 | 300 |
| 1AQL | 3.01.01.0013 | a/b | A | 532 | 1997 | 2.8 | 0.211 | 300 |
| 1AST | 3.04.24.0021 | a+b | A | 200 | 1993 | 1.8 | 0.158 | 300 |
| 1B04 | 6.05.01.0002 | a+b | A | 310 | 1998 | 2.8 | 0.23 | 300 |
| 1B2R | 1.18.01.0002 | b, a/b | A | 295 | 1998 | 1.8 | 0.19 | 300 |
| 1BBS | 3.04.23.0015 | b | A | 325 | 1992 | 2.8 | 0.196 | 300 |
| 1BF2 | 3.02.01.0068 | b, b, a/b | A | 750 | 1998 | 2 | 0.161 | 300 |
| 1BG0 | 2.07.03.0003 | a, a+b | A | 356 | 1998 | 1.86 | 0.196 | 300 |
| 1BH2 | na | a, a/b | A | 315 | 1998 | 2.1 | 0.19 | 300 |
| 1BOL | 2.07.01.0068 | a+b | A | 222 | 1998 | 2 | 0.185 | 300 |
| 1BP2 | 3.01.01.0004 | a | A | 123 | 1981 | 1.7 | 0.171 | 300 |
| 1BQC | 3.02.01.0078 | a/b | A | 302 | 1998 | 1.5 | 0.119 | 300 |
| 1BS0 | 2.03.01.0047 | a/b | A | 383 | 1998 | 1.65 | 0.178 | 300 |
| 1BS4 | 3.05.01.0088 | a+b | A | 168 | 1998 | 1.9 | 0.193 | 300 |
| 1BS9 | 3.01.01.0006 | a/b | A | 207 | 1998 | 1.1 | 0.123 | 300 |
| 1BT1 | 1.10.03.0001 | a | A | 336 | 1998 | 2.7 | 0.168 | 300 |
| 1BU7 | 1.06.02.0004 | a | A | 455 | 1998 | 1.65 | 0.197 | 300 |
| 1BVV | 3.02.01.0008 | b | A | 185 | 1998 | 1.8 | 0.189 | 300 |
| 1BVZ | 3.02.01.0135 | b, b, a/b | A | 585 | 1998 | 2.6 | 0.196 | 300 |
| 1BWL | 1.06.99.0001 | a/b | A | 399 | 1998 | 2.7 | 0.19 | 300 |
| 1BWZ | 5.01.01.0007 | a+b, a+b | A | 274 | 1998 | 2.72 | 0.19 | 300 |
| 1BYA | 3.02.01.0002 | a/b | A | 491 | 1994 | 2.2 | 0.169 | 137 |
| 1BZC | 3.01.03.0048 | a/b | A | 297 | 1998 | 2.35 | 0.204 | 300 |
| 1C82 | 4.02.02.0001 | a, b, b | A | 719 | 2000 | 1.7 | 0.215 | 244 |
| 1CA2 | 4.02.01.0001 | b | A | 256 | 1989 | 2 | 0.173 | 300 |
| 1CB8 | 4.02.02.0005 | a, b, b | A | 674 | 1999 | 1.9 | 0.195 | 240 |
| 1CBX | 3.04.17.0001 | a/b | A | 307 | 1991 | 2 | 0.166 | 300 |

| | | | | | | | | |
|---|---|---|---|---|---|---|---|---|
| 1CDG | 2.04.01.0019 | b, b, b, a/b | A | 686 | 1993 | 2 | 0.164 | 300 |
| 1CEL | 3.02.01.0091 | b | A | 433 | 1994 | 1.8 | 0.181 | 239 |
| 1CHD | 3.01.01.0061 | a/b | A | 198 | 1995 | 1.75 | 0.182 | 300 |
| 1CHK | 3.02.01.0132 | a+b | B | 238 | 1995 | 2.4 | 0.181 | 73 |
| 1CM0 | 2.03.01.0048 | a+b | A | 162 | 1999 | 2.3 | 0.223 | 300 |
| 1CNS | 3.04.23.0004 | a+b | A | 243 | 1995 | 1.91 | 0.186 | 300 |
| 1COY | 1.01.03.0006 | a/b, a+b | A | 501 | 1993 | 1.8 | 0.159 | 300 |
| 1CQQ | 2.07.07.0048 | b | A | 180 | 1999 | 1.85 | 0.218 | 194 |
| 1CVR | 3.04.22.0037 | b, a/b | A | 432 | 1999 | 2 | 0.163 | 134 |
| 1CWY | 2.04.01.0025 | a/b | A | 500 | 1999 | 2 | 0.19 | 300 |
| 1CZ1 | 3.02.01.0058 | a/b | A | 394 | 1999 | 1.85 | 0.166 | 300 |
| 1CZF | 3.02.01.0015 | b | A | 335 | 1999 | 1.68 | 0.166 | 300 |
| 1D1Q | 3.01.03.0002 | a/b | A | 159 | 1999 | 1.7 | 0.17 | 300 |
| 1D4C | 1.03.99.0001 | a, a/b, a+b | A | 570 | 1999 | 2.9 | 0.21 | 300 |
| 1D6O | 5.02.01.0008 | a+b | A | 107 | 1999 | 1.85 | 0.192 | 300 |
| 1DDJ | 3.04.21.0007 | b | A | 247 | 1999 | 2 | 0.194 | 300 |
| 1DGK | 2.07.01.0001 | a/b, a/b, a/b, a/b | N | 898 | 1999 | 2.8 | 0.258 | 60 |
| 1DGS | 6.05.01.0002 | a, b, a+b | A | 581 | 1999 | 2.9 | 0.228 | 300 |
| 1DIN | 3.01.01.0045 | a/b | A | 233 | 1996 | 1.8 | 0.15 | 300 |
| 1DNP | 4.01.99.0003 | a, a/b | A | 469 | 1995 | 2.3 | 0.172 | 300 |
| 1DVE | 1.14.99.0003 | a | A | 214 | 2000 | 2.4 | 0.214 | 300 |
| 1E0C | 2.08.01.0001 | a/b, a/b | A | 270 | 2000 | 1.8 | 0.18 | 300 |
| 1E1A | 3.01.08.0002 | b | A | 312 | 2000 | 1.8 | 0.1763 | 300 |
| 1E6E | 1.18.01.0002 | a/b, a/b | A | 457 | 2000 | 2.3 | 0.222 | 300 |
| 1EB6 | 3.04.24.0039 | a+b | A | 177 | 2001 | 1 | 0.104 | 225 |
| 1EH5 | 3.01.02.0022 | a/b | A | 279 | 2000 | 2.5 | 0.226 | 300 |
| 1EUG | 3.02.02.0003 | a/b | A | 225 | 1998 | 1.6 | 0.194 | 300 |
| 1EUU | 3.02.01.0018 | b, b, b | A | 601 | 1996 | 2.5 | 0.228 | 300 |
| 1EX1 | 3.02.01.0058 | a/b, a/b | A | 602 | 1998 | 2.2 | 0.17 | 300 |
| 1EXN | 3.01.11.0003 | a, a/b | A | 267 | 1997 | 2.5 | 0.227 | 300 |
| 1EXP | 3.02.01.0008 | a/b | A | 312 | 1996 | 1.8 | 0.208 | 300 |
| 1EYP | 5.05.01.0006 | a+b | A | 212 | 2000 | 2.5 | 0.249 | 272 |
| 1FF3 | 1.08.04.0006 | a+b | A | 210 | 2000 | 1.9 | 0.195 | 300 |
| 1FGH | 4.02.01.0003 | a/b, a/b | A | 753 | 1996 | 2.05 | 0.177 | 300 |
| 1FHL | 3.02.01.0089 | a/b | A | 334 | 2000 | 2.3 | 0.166 | 300 |
| 1FOA | 2.04.01.0101 | a/b | A | 342 | 2000 | 1.8 | 0.185 | 200 |
| 1G24 | 2.04.02.0000 | a+b | A | 211 | 2000 | 1.7 | 0.24 | 241 |
| 1G6T | 2.05.01.0019 | a+b | A | 427 | 2000 | 1.6 | 0.155 | 300 |
| 1G8O | 2.04.01.0087 | a/b | A | 287 | 2000 | 2.3 | 0.274 | 98 |
| 1GA8 | 2.04.01.0044 | a/b | A | 271 | 2000 | 2 | 0.189 | 300 |
| 1GAL | 1.01.03.0004 | a/b, a+b | A | 581 | 1992 | 2.3 | 0.181 | 300 |
| 1GCU | 1.03.01.0024 | a/b, a+b | A | 292 | 2000 | 1.4 | 0.219 | 300 |
| 1GOG | 1.01.03.0009 | b, b, b | A | 639 | 1993 | 1.9 | 0.17 | 300 |
| 1GP5 | 1.14.11.0019 | b | A | 346 | 2001 | 2.2 | 0.204 | 300 |
| 1GQ8 | 3.01.01.0011 | b | A | 318 | 2001 | 1.75 | 0.181 | 300 |
| 1H19 | 3.03.02.0006 | a, b, a+b | A | 610 | 2002 | 2.1 | 0.184 | 300 |

| | | | | | | | | |
|---|---|---|---|---|---|---|---|---|
| 1H3I | 2.01.01.0043 | b, b | A | 293 | 2002 | 2.1 | 0.21 | 300 |
| 1HKA | 2.07.06.0003 | a+b | A | 158 | 1998 | 1.5 | 0.182 | 300 |
| 1HPL | 3.01.01.0003 | b, a/b | A | 448 | 1993 | 2.3 | 0.159 | 300 |
| 1HPM | 3.06.01.0003 | a/b, a/b | A | 378 | 1995 | 1.7 | 0.205 | 300 |
| 1HQC | 3.06.01.0003 | a, a/b | A | 314 | 2000 | 3.2 | 0.263 | 300 |
| 1I1E | 3.04.24.0069 | b, b, a+b, Coiled coil proteins | A | 1287 | 2001 | 2.5 | 0.217 | 32 |
| 1I1I | 3.04.24.0016 | a+b | P | 665 | 2001 | 2.3 | 0.224 | 300 |
| 1I9A | 5.03.03.0002 | a+b | A | 175 | 2001 | 2.5 | 0.229 | 300 |
| 1IDJ | 4.02.02.0010 | b | A | 359 | 1996 | 2.4 | 0.16 | 300 |
| 1IG8 | 2.07.01.0001 | a/b, a/b | A | 469 | 2001 | 2.2 | 0.162 | 300 |
| 1IM5 | 3.05.01.0019 | a/b | A | 179 | 2001 | 1.65 | 0.152 | 300 |
| 1INP | 3.01.03.0057 | multi-domain | A | 400 | 1994 | 2.3 | 0.198 | 300 |
| 1IU4 | 2.03.02.0013 | a+b | A | 331 | 2002 | 2.4 | 0.199 | 8 |
| 1J00 | 3.01.02.0000 | a/b | A | 176 | 2002 | 2 | 0.239 | 300 |
| 1J09 | 6.01.01.0017 | a, a/b | A | 468 | 2002 | 1.8 | 0.199 | 300 |
| 1JH6 | 3.01.04.0000 | a+b | A | 181 | 2001 | 1.8 | 0.192 | 282 |
| 1JMS | 2.07.07.0031 | a, a, a+b | A | 360 | 2001 | 2.36 | 0.214 | 300 |
| 1JOA | 1.11.01.0001 | a/b, a/b, a+b | A | 446 | 1996 | 2.8 | 0.179 | 300 |
| 1JS4 | 3.02.01.0004 | a, b | A | 605 | 1997 | 2 | 0.199 | 300 |
| 1K30 | 2.03.01.0015 | a/b | A | 363 | 2001 | 1.9 | 0.186 | 48 |
| 1KL7 | 4.02.03.0001 | a/b | A | 503 | 2001 | 2.7 | 0.201 | 300 |
| 1KNP | 1.04.03.0016 | a, a/b, a+b | A | 529 | 2001 | 2.6 | 0.23 | 300 |
| 1L1D | 1.08.04.0006 | b | A | 142 | 2002 | 1.85 | 0.207 | 300 |
| 1L1L | 1.17.04.0002 | a/b | A | 717 | 2002 | 1.75 | 0.223 | 300 |
| 1L6P | 1.08.01.0008 | b | A | 121 | 2002 | 1.65 | 0.141 | 300 |
| 1L7N | 3.01.03.0003 | a/b | A | 207 | 2002 | 1.8 | 0.191 | 300 |
| 1L7Q | 3.01.01.0001 | b, a/b | A | 571 | 2002 | 1.76 | 0.183 | 300 |
| 1L8T | 2.07.01.0095 | a+b | A | 263 | 2002 | 2.4 | 0.234 | 300 |
| 1L9X | 3.04.19.0009 | a/b | A | 288 | 2002 | 1.6 | 0.181 | 300 |
| 1LBA | 3.05.01.0028 | a+b | A | 146 | 1993 | 2.2 | 0.19 | 300 |
| 1LBU | 3.04.17.0008 | a, a+b | A | 213 | 1996 | 1.8 | 0.164 | 300 |
| 1LDM | 1.01.01.0027 | a/b, a+b | A | 329 | 1987 | 2.1 | 0.173 | 300 |
| 1LJL | 1.03.99.0001 | a/b | A | 130 | 2002 | 2.01 | 0.214 | 300 |
| 1LVH | 5.04.02.0006 | a/b | A | 220 | 2002 | 2.3 | 0.243 | 300 |
| 1M53 | 5.04.99.0011 | b, a/b | A | 556 | 2002 | 2.2 | 0.194 | 300 |
| 1MBB | 1.01.01.0158 | a+b, a+b | A | 340 | 1995 | 2.3 | 0.238 | 300 |
| 1MJ9 | 2.03.01.0048 | a+b | A | 273 | 2002 | 2.5 | 0.232 | 300 |
| 1MLA | 2.03.01.0039 | a/b, a+b | A | 305 | 1995 | 1.5 | 0.184 | 300 |
| 1MRQ | 1.03.01.0020 | a/b | A | 323 | 2002 | 1.59 | 0.173 | 300 |
| 1MUD | 3.02.02.0000 | a | A | 225 | 1998 | 1.8 | 0.192 | 300 |
| 1MUG | 3.02.02.0000 | a/b | A | 165 | 1998 | 1.8 | 0.198 | 300 |
| 1NDH | 1.06.02.0002 | b, a/b | A | 270 | 1994 | 2.1 | 0.223 | 300 |
| 1NDI | 2.03.01.0007 | a/b, a/b | A | 596 | 2002 | 2.3 | 0.27 | 300 |
| 1NLU | 3.04.21.0100 | a/b | A | 368 | 2003 | 1.3 | 0.164 | 300 |
| 1O8A | 3.04.15.0001 | a+b | A | 574 | 2002 | 2 | 0.18 | 300 |

| | | | | | | | | |
|---|---|---|---|---|---|---|---|---|
| 1O98 | 5.04.02.0001 | a/b, a/b | A | 509 | 2002 | 1.4 | 0.19 | 300 |
| 1OBA | 3.02.01.0017 | b, a/b | A | 338 | 2003 | 2.45 | 0.205 | 300 |
| 1OG1 | 2.04.02.0031 | a+b | A | 223 | 2003 | 2 | 0.202 | 165 |
| 1OGO | 3.02.01.0011 | b, b | X | 572 | 2003 | 1.65 | 0.188 | 161 |
| 1OJ4 | 2.07.01.0148 | a+b, a+b | A | 277 | 2003 | 2.01 | 0.1655 | 300 |
| 1ONR | 2.02.01.0002 | a/b | A | 316 | 1996 | 1.87 | 0.201 | 300 |
| 1OPM | 1.14.17.0003 | b, b | A | 310 | 1999 | 2.1 | 0.2 | 275 |
| 1OXA | 1.00.00.0000 | a | A | 403 | 1995 | 2.1 | 0.196 | 300 |
| 1OYG | 2.04.01.0010 | b | A | 440 | 2003 | 1.5 | 0.165 | 114 |
| 1P1X | 4.01.02.0004 | a/b | A | 250 | 2003 | 0.99 | 0.143 | 300 |
| 1P3D | 6.03.02.0008 | a/b, a/b, a/b | A | 449 | 2003 | 1.7 | 0.168 | 300 |
| 1P5D | 5.04.02.0008 | a/b, a/b, a/b, a+b | X | 454 | 2003 | 1.6 | 0.156 | 300 |
| 1PA9 | 3.01.03.0048 | a/b | A | 279 | 2003 | 2 | 0.226 | 300 |
| 1PII | 4.01.01.0048 | a/b, a/b | A | 452 | 1991 | 2 | 0.173 | 286 |
| 1PKN | 2.07.01.0040 | b, a/b, a/b | A | 514 | 1994 | 2.9 | 0.191 | 300 |
| 1PMI | 5.03.01.0008 | b | A | 440 | 1996 | 1.7 | 0.184 | 300 |
| 1PP4 | 3.01.01.0000 | a/b | A | 233 | 2003 | 2.5 | 0.182 | 300 |
| 1PS9 | 1.03.01.0034 | a/b, a/b, a/b | A | 671 | 2003 | 2.2 | 0.203 | 300 |
| 1PTD | 4.06.01.0013 | a/b | A | 296 | 1995 | 2.6 | 0.187 | 300 |
| 1PUD | 2.04.02.0029 | a/b | A | 372 | 1996 | 1.85 | 0.19 | 300 |
| 1PYL | 3.01.04.0008 | a+b | A | 96 | 2003 | 1.51 | 0.149 | 223 |
| 1Q91 | 3.01.03.0005 | a/b | A | 194 | 2003 | 1.6 | 0.186 | 300 |
| 1QAM | 2.01.01.0048 | a/b | A | 235 | 1999 | 2.2 | 0.221 | 300 |
| 1QAZ | 3.05.01.0045 | a | A | 351 | 1999 | 1.78 | 0.18 | 300 |
| 1QBA | 3.02.01.0052 | b, b, a/b, a+b | A | 858 | 1996 | 1.85 | 0.139 | 300 |
| 1QFM | 3.04.21.0026 | b, a/b | A | 705 | 1999 | 1.4 | 0.19 | 300 |
| 1QGX | 3.01.03.0007 | multi-domain | A | 354 | 1999 | 1.6 | 0.197 | 300 |
| 1QJE | 1.21.03.0001 | b | A | 327 | 1999 | 1.35 | 0.131 | 300 |
| 1QK2 | 3.02.01.0091 | a/b | A | 363 | 1999 | 2 | 0.189 | 295 |
| 1QV0 | 1.13.12.0005 | a | A | 185 | 2003 | 1.1 | 0.134 | 300 |
| 1QWN | 3.02.01.0114 | a, b, a/b | A | 1014 | 2003 | 1.2 | 0.175 | 300 |
| 1R1J | 3.04.24.0011 | a+b | A | 696 | 2003 | 2.35 | 0.222 | 300 |
| 1R44 | 3.04.13.0000 | a+b | A | 202 | 2003 | 2.25 | 0.254 | 300 |
| 1R4Z | 3.01.01.0003 | a/b | A | 179 | 2003 | 1.8 | 0.177 | 300 |
| 1RBN | 3.01.27.0005 | a+b | A | 124 | 1994 | 2.1 | 0.166 | 292 |
| 1RDD | 3.01.26.0004 | a/b | A | 155 | 1993 | 2.8 | 0.19 | 300 |
| 1RU4 | 4.02.02.0002 | b | A | 400 | 2003 | 1.6 | 0.167 | 300 |
| 1S3I | 1.05.01.0006 | b, a/b | A | 307 | 2004 | 2.3 | 0.242 | 300 |
| 1S95 | 3.01.03.0016 | a+b | A | 324 | 2004 | 1.6 | 0.169 | 300 |
| 1SCA | 3.04.21.0062 | a/b | A | 274 | 1993 | 2 | 0.156 | 300 |
| 1SLL | 4.02.02.0015 | b, b | A | 679 | 1997 | 2 | 0.167 | 184 |
| 1SML | 3.05.02.0006 | a+b | A | 266 | 1998 | 1.7 | 0.18 | 300 |
| 1SNZ | 5.01.03.0003 | b | A | 344 | 2004 | 2.2 | 0.172 | 300 |
| 1TAH | 3.01.01.0003 | a/b | B | 318 | 1993 | 3 | 0.159 | 300 |
| 1TLP | 3.04.24.0027 | a+b | E | 316 | 1987 | 2.3 | 0.174 | 300 |
| 1U3F | 6.03.03.0002 | a/b | A | 164 | 2004 | 2.5 | 0.223 | 300 |

| | | | | | | | | |
|---|---|---|---|---|---|---|---|---|
| 1U5U | 4.02.01.0092 | multi-domain | A | 366 | 2004 | 2 | 0.196 | 300 |
| 1UAS | 3.02.01.0022 | b, a/b | A | 362 | 2003 | 1.5 | 0.16 | 300 |
| 1UN1 | 2.04.01.0207 | b | A | 267 | 2003 | 2.1 | 0.206 | 300 |
| 1VID | 2.01.01.0006 | a/b | A | 213 | 1996 | 2 | 0.194 | 300 |
| 1VLB | 1.02.99.0007 | a, a+b, a+b, a+b | A | 907 | 2004 | 1.28 | 0.148 | 300 |
| 1VNC | 1.11.01.0010 | a | A | 576 | 1995 | 2.1 | 0.2 | 126 |
| 1VQ1 | 2.01.01.0000 | a/b | A | 263 | 2004 | 2.8 | 0.208 | 300 |
| 1W0H | 3.01.00.0000 | a/b | A | 194 | 2004 | 1.59 | 0.168 | 300 |
| 1XQW | 3.04.11.0005 | na | A | 290 | 2004 | 2 | 0.248 | 300 |
| 1Y9M | 3.02.01.0080 | b, b | A | 517 | 2004 | 1.89 | 0.165 | 300 |
| 1YON | 1.01.01.0169 | a, a/b | A | 292 | 2005 | 1.95 | 0.166 | 300 |
| 1YSC | 3.04.16.0005 | a/b | A | 421 | 1994 | 2.8 | 0.162 | 300 |
| 1ZE1 | 4.02.01.0070 | b, a+b | A | 308 | 2005 | 2.9 | 0.235 | 300 |
| 1ZIO | 2.07.04.0003 | a/b, Small proteins | A | 217 | 1996 | 1.96 | 0.161 | 300 |
| 206L | 3.02.01.0017 | a+b | A | 162 | 1996 | 1.75 | 0.17 | 300 |
| 2A0N | 4.01.03.0000 | a/b | A | 251 | 2005 | 1.64 | 0.167 | 300 |
| 2ABK | 4.02.99.0018 | a | A | 211 | 1995 | 1.85 | 0.185 | 300 |
| 2ACY | 3.06.01.0007 | a+b | A | 98 | 1996 | 1.8 | 0.179 | 300 |
| 2AYH | 3.02.01.0073 | b | A | 214 | 1995 | 1.6 | 0.143 | 300 |
| 2CPO | 1.11.01.0010 | a, a | A | 298 | 1996 | 2.1 | 0.186 | 158 |
| 2CPU | 3.02.01.0001 | b, a/b | A | 495 | 1999 | 2 | 0.167 | 300 |
| 2DLN | 6.03.02.0004 | a/b, a+b | A | 306 | 1994 | 2.3 | 0.172 | 300 |
| 2EBN | 3.02.01.0096 | a/b | A | 285 | 1994 | 2 | 0.158 | 300 |
| 2ENG | 3.02.01.0004 | b | A | 205 | 1995 | 1.5 | 0.105 | 120 |
| 2F61 | 3.02.01.0045 | b, a/b | A | 497 | 2005 | 2.5 | 0.196 | 300 |
| 2F9R | 3.01.04.0041 | na | A | 285 | 2005 | 1.85 | 0.187 | 174 |
| 2NLR | 3.02.01.0004 | b | A | 222 | 1998 | 1.2 | 0.112 | 259 |
| 2NMT | 2.03.01.0097 | a+b, a+b | A | 422 | 1998 | 2.9 | 0.228 | 181 |
| 2PEC | 4.02.02.0002 | b | A | 352 | 1994 | 2.2 | 0.18 | 300 |
| 2PIA | 1.00.00.0000 | b, a/b, a+b | A | 321 | 1993 | 2 | 0.186 | 300 |
| 2PTH | 3.01.01.0029 | a/b | A | 193 | 1997 | 1.2 | 0.196 | 300 |
| 2SQC | 5.04.99.0017 | a, a | A | 623 | 1998 | 2 | 0.153 | 300 |
| 2THI | 2.05.01.0002 | a/b | A | 362 | 1998 | 2.5 | 0.195 | 300 |
| 2TPS | 2.05.01.0003 | a/b | A | 226 | 1999 | 1.25 | 0.181 | 300 |
| 5EAT | 4.02.03.0009 | a, a | A | 532 | 1997 | 2.8 | 0.24 | 300 |
| 7ATJ | 1.11.01.0007 | a | A | 305 | 1999 | 1.47 | 0.16 | 300 |

[1] Number of sites in each protein sequence

[2] Number of sequence in the MSA profile of each protein

**TABLE S2**

**Comparison between sequence variability measures using RSA as reference**

| ref. | x1 | x2 | Δ[1] | T[2] | Paired.T[3] | %[4] | Binomial[5] | d[6] | W[7] | Paired.W[8] |
|---|---|---|---|---|---|---|---|---|---|---|
| RSA[T] | *CS* | *ET* | 0.006 | 1.3 | 0.096628 | 51.9 | 0.316985 | 0.002 | 12249 | 0.282008 |
| | *CS* | *KBSP* | 0.050 | 8.3 | 5.31E-15 | 72.7 | 9.26E-12 | 0.059 | 18573 | 4.53E-14 |
| | *CS* | *VTSP* | 0.051 | 8.4 | 2.44E-15 | 73.6 | 1.23E-12 | 0.060 | 18697 | 1.62E-14 |
| | *CS* | *EN* | 0.194 | 18.1 | 2.25E-45 | 95.4 | 4.92E-49 | 0.186 | 23230 | 2.97E-36 |
| | *ET* | *KBSP* | 0.044 | 8.1 | 1.66E-14 | 74.1 | 4.32E-13 | 0.057 | 18862 | 3.98E-15 |
| | *ET* | *VTSP* | 0.045 | 8.4 | 3.60E-15 | 74.1 | 4.32E-13 | 0.058 | 18965 | 1.64E-15 |
| | *ET* | *EN* | 0.188 | 17.3 | 7.33E-43 | 94.0 | 2.51E-45 | 0.184 | 23118 | 1.37E-35 |
| | *KBSP* | *VTSP* | 0.001 | 2.0 | 0.021100 | 50.9 | 0.419158 | 0.001 | 12196 | 0.301792 |
| | *KBSP* | *EN* | 0.144 | 12.8 | 1.47E-28 | 84.7 | 1.10E-26 | 0.127 | 21986 | 3.02E-29 |
| | *VTSP* | *EN* | 0.143 | 12.6 | 5.78E-28 | 84.7 | 1.10E-26 | 0.127 | 21862 | 1.36E-28 |

1 Difference between the mean correlations of ref with x1 and x2

2 T-value derived by T test with paired variables

3 P-value derived by T test with paired variables for x1 > x2

4 The proportion of cases for which x1 > x2

5 P-value derived by Binomial test for x1 > x2

6 Difference between the median correlations of ref with x1 and x2

7 W-value derived by Wilcoxon matched-paired signed-rank test

8 P-value derived by Wilcoxon matched-paired signed-rank test for x1 > x2

**Table S3**
**Comparison between sequence variability measures using WCN as reference**

| ref. | x1 | x2 | Δ[1] | T[2] | Paired.T[3] | %[4] | Binomial[5] | d[6] | W[7] | Paired.W[8] |
|---|---|---|---|---|---|---|---|---|---|---|
| WCN | *CS* | *ET* | 0.000 | 0.0 | 0.514733 | 42.6 | 0.987740 | -0.001 | 10306 | 0.937732 |
| | *CS* | *KBSP* | 0.041 | 6.5 | 3.53E-10 | 68.5 | 2.72E-08 | 0.052 | 17360 | 4.26E-10 |
| | *CS* | *VTSP* | 0.041 | 6.4 | 4.25E-10 | 69.4 | 5.37E-09 | 0.053 | 17338 | 4.95E-10 |
| | *CS* | *EN* | 0.196 | 15.9 | 1.63E-38 | 93.5 | 3.66E-44 | 0.175 | 22976 | 9.29E-35 |
| | *ET* | *KBSP* | 0.041 | 6.4 | 3.84E-10 | 72.7 | 9.26E-12 | 0.053 | 17931 | 7.11E-12 |
| | *ET* | *VTSP* | 0.042 | 6.5 | 3.31E-10 | 72.2 | 2.46E-11 | 0.054 | 17858 | 1.22E-11 |
| | *ET* | *EN* | 0.196 | 15.5 | 2.45E-37 | 93.1 | 4.96E-43 | 0.176 | 22799 | 9.77E-34 |
| | *KBSP* | *VTSP* | 0.000 | 0.1 | 0.461592 | 40.3 | 0.998329 | 0.001 | 9805 | 0.981275 |
| | *KBSP* | *EN* | 0.154 | 11.9 | 1.04E-25 | 86.1 | 5.56E-29 | 0.124 | 21810 | 2.55E-28 |
| | *VTSP* | *EN* | 0.154 | 11.8 | 1.52E-25 | 83.3 | 1.59E-24 | 0.123 | 21715 | 7.96E-28 |

[1] Difference between the mean correlations of ref with x1 and x2
[2] T-value derived by T test with paired variables
[3] P-value derived by T test with paired variables for x1 > x2
[4] The proportion of cases for which x1 > x2
[5] P-value derived by Binomial test for x1 > x2
[6] Difference between the median correlations of ref with x1 and x2
[7] W-value derived by Wilcoxon matched-paired signed-rank test
[8] P-value derived by Wilcoxon matched-paired signed-rank test for x1 > x2

**TABLE S4**

**Comparison between SA profiles using sequence variability measures as reference**

| ref. | x1 | x2 | Δ[1] | T[2] | Paired.T[3] | %[4] | Binomial[5] | d[6] | W[7] | Paired.W[8] |
|---|---|---|---|---|---|---|---|---|---|---|
| *CS* | RSA$^T$ | RSA$^M$ | 0.001 | 3.5 | 3.34E-04 | 63.9 | 2.69E-05 | 0.006 | 15267 | 5.70E-05 |
| | RSA$^T$ | RSA$^R$ | 0.001 | 3.4 | 4.21E-04 | 65.3 | 4.19E-06 | 0.005 | 15111 | 1.13E-04 |
| | RSA$^T$ | ASA | 0.008 | 4.2 | 1.83E-05 | 60.2 | 1.67E-03 | 0.009 | 15375 | 3.50E-05 |
| | RSA$^M$ | RSA$^R$ | 0.000 | 0.1 | 0.470690 | 47.2 | 0.811776 | -0.001 | 10876 | 0.820210 |
| | RSA$^M$ | ASA | 0.007 | 3.3 | 5.73E-04 | 60.2 | 1.67E-03 | 0.002 | 14615 | 8.17E-04 |
| | RSA$^R$ | ASA | 0.007 | 3.3 | 5.69E-04 | 59.3 | 3.91E-03 | 0.004 | 14688 | 6.21E-04 |
| *ET* | RSA$^T$ | RSA$^M$ | 0.001 | 3.5 | 2.63E-04 | 61.1 | 6.65E-04 | 0.000 | 14946 | 2.24E-04 |
| | RSA$^T$ | RSA$^R$ | 0.001 | 3.8 | 1.06E-04 | 60.2 | 1.67E-03 | 0.002 | 14949 | 2.22E-04 |
| | RSA$^T$ | ASA | 0.011 | 6.0 | 4.85E-09 | 65.3 | 4.19E-06 | 0.014 | 17033 | 3.75E-09 |
| | RSA$^M$ | RSA$^R$ | 0.000 | 1.0 | 0.162618 | 48.6 | 0.683015 | 0.002 | 12013 | 0.374389 |
| | RSA$^M$ | ASA | 0.009 | 4.9 | 8.73E-07 | 62.0 | 2.46E-04 | 0.014 | 16322 | 2.78E-07 |
| | RSA$^R$ | ASA | 0.009 | 4.8 | 1.28E-06 | 62.5 | 1.45E-04 | 0.013 | 16251 | 4.14E-07 |
| *KBSP* | RSA$^T$ | RSA$^M$ | 0.003 | 9.6 | 1.25E-18 | 77.3 | 1.46E-16 | 0.003 | 19443 | 2.23E-17 |
| | RSA$^T$ | RSA$^R$ | 0.003 | 9.5 | 2.03E-18 | 75.9 | 5.21E-15 | 0.003 | 19497 | 1.35E-17 |
| | RSA$^T$ | ASA | 0.012 | 6.4 | 4.24E-10 | 68.1 | 5.94E-08 | 0.008 | 17530 | 1.31E-10 |
| | RSA$^M$ | RSA$^R$ | 0.000 | -0.3 | 0.632773 | 45.4 | 0.923561 | 0.000 | 11168 | 0.725292 |
| | RSA$^M$ | ASA | 0.009 | 4.5 | 6.13E-06 | 64.4 | 1.48E-05 | 0.005 | 16211 | 5.16E-07 |
| | RSA$^R$ | ASA | 0.009 | 4.5 | 4.91E-06 | 63.0 | 8.44E-05 | 0.005 | 16214 | 5.08E-07 |
| *VTSP* | RSA$^T$ | RSA$^M$ | 0.003 | 9.8 | 3.12E-19 | 77.3 | 1.46E-16 | 0.003 | 19549 | 8.32E-18 |
| | RSA$^T$ | RSA$^R$ | 0.003 | 9.7 | 5.49E-19 | 76.9 | 4.94E-16 | 0.004 | 19647 | 3.30E-18 |
| | RSA$^T$ | ASA | 0.012 | 6.4 | 5.84E-10 | 67.1 | 2.66E-07 | 0.008 | 17500 | 1.62E-10 |
| | RSA$^M$ | RSA$^R$ | 0.000 | -0.4 | 0.641680 | 45.4 | 0.923561 | 0.000 | 11122 | 0.741720 |
| | RSA$^M$ | ASA | 0.008 | 4.4 | 8.54E-06 | 63.9 | 2.69E-05 | 0.005 | 16149 | 7.25E-07 |
| | RSA$^R$ | ASA | 0.008 | 4.5 | 6.84E-06 | 62.5 | 1.45E-04 | 0.004 | 16173 | 6.36E-07 |
| *EN* | ASA | RSA$^T$ | 0.006 | 3.1 | 1.01E-03 | 62.5 | 1.45E-04 | 0.011 | 15025 | 1.62E-04 |
| | ASA | RSA$^M$ | 0.007 | 3.5 | 3.09E-04 | 63.4 | 4.81E-05 | 0.015 | 15416 | 2.90E-05 |
| | ASA | RSA$^R$ | 0.007 | 3.5 | 2.44E-04 | 64.4 | 1.48E-05 | 0.014 | 15456 | 2.41E-05 |
| | RSA$^T$ | RSA$^M$ | 0.001 | 3.0 | 1.73E-03 | 64.8 | 7.93E-06 | 0.004 | 14982 | 1.93E-04 |
| | RSA$^T$ | RSA$^R$ | 0.001 | 3.3 | 5.31E-04 | 64.8 | 7.93E-06 | 0.003 | 15345 | 4.01E-05 |
| | RSA$^M$ | RSA$^R$ | 0.000 | 1.1 | 0.141269 | 54.2 | 0.123666 | -0.001 | 12503 | 0.196803 |

[1] Difference between the mean correlations of ref with x1 and x2

[2] T-value derived by T test with paired variables

[3] P-value derived by T test with paired variables for x1 > x2

[4] The proportion of cases for which x1 > x2

[5] P-value derived by Binomial test for x1 > x2

[6] Difference between the median correlations of ref with x1 and x2

[7] W-value derived by Wilcoxon matched-paired signed-rank test

[8] P-value derived by Wilcoxon matched-paired signed-rank test for x1 > x2

**TABLE S5**

**Mean Pearson correlations between $CN_{cut\text{-}off}$ and sequence profiles**

| Profile | CS | ET | KBSP | VTSP | EN |
|---|---|---|---|---|---|
| **$CN_9$** | 0.5324 | 0.5277 | 0.4913 | 0.4909 | 0.3617 |
| **$CN_{10}$** | 0.5589 | 0.5513 | 0.5126 | 0.5123 | 0.3847 |
| **$CN_{11}$** | 0.5648 | 0.5565 | 0.5233 | 0.5233 | 0.3846 |
| **$CN_{12}$** | 0.5768 | 0.5695 | 0.5321 | 0.5321 | 0.3959 |
| **$CN_{13}$** | 0.5820 | 0.5765 | 0.5383 | 0.5383 | 0.4004 |
| **$CN_{14}$** | 0.5859 | 0.5818 | 0.5430 | 0.5430 | 0.4054 |
| **$CN_{15}$** | 0.5862 | 0.5832 | 0.5449 | 0.5450 | 0.4073 |
| **$CN_{16}$** | 0.5888 | 0.5867 | 0.5474 | 0.5476 | 0.4113 |
| **$CN_{17}$** | 0.5918 | 0.5905 | 0.5491 | 0.5493 | 0.4155 |
| **$CN_{18}$** | 0.5951 | 0.5945 | 0.5506 | 0.5508 | 0.4198 |
| **$CN_{19}$** | 0.5958 | 0.5956 | 0.5500 | 0.5502 | 0.4217 |
| **$CN_{20}$** | 0.5952 | 0.5954 | 0.5484 | 0.5485 | 0.4223 |
| **$CN_{21}$** | 0.5948 | 0.5953 | 0.5468 | 0.5469 | 0.4215 |
| **$CN_{22}$** | 0.5933 | 0.5942 | 0.5444 | 0.5445 | 0.4195 |
| **$CN_{23}$** | 0.5898 | 0.5914 | 0.5409 | 0.5410 | 0.4157 |
| **$CN_{24}$** | 0.5864 | 0.5884 | 0.5368 | 0.5368 | 0.4116 |
| **$CN_{25}$** | 0.5815 | 0.5838 | 0.5316 | 0.5316 | 0.4056 |
| **$CN_{26}$** | 0.5768 | 0.5793 | 0.5265 | 0.5264 | 0.3999 |
| **$CN_{27}$** | 0.5706 | 0.5735 | 0.5202 | 0.5201 | 0.3927 |
| **$CN_{28}$** | 0.5639 | 0.5672 | 0.5138 | 0.5136 | 0.3848 |
| **$CN_{29}$** | 0.5566 | 0.5601 | 0.5063 | 0.5061 | 0.3766 |
| **$CN_{30}$** | 0.5483 | 0.5520 | 0.4984 | 0.4981 | 0.3675 |

**TABLE S6**

**Comparison between LPD profiles using sequence variability measures as reference**

| ref. | x1 | x2 | Δ[1] | T[2] | Paired.T[3] | %[4] | Binomial[5] | d[6] | W[7] | Paired.W[8] |
|---|---|---|---|---|---|---|---|---|---|---|
| *CS* | | | 0.013 | 5.8 | 1.51E-08 | 67.1 | 2.66E-07 | 0.015 | 16954 | 6.23E-09 |
| *ET* | | | 0.013 | 5.7 | 2.02E-08 | 64.4 | 1.48E-05 | 0.014 | 16730 | 2.52E-08 |
| *KBSP* | WCN | CN* | 0.017 | 8.0 | 4.52E-14 | 70.4 | 9.73E-10 | 0.023 | 18269 | 5.27E-13 |
| *VTSP* | | | 0.016 | 7.9 | 9.44E-14 | 70.8 | 4.01E-10 | 0.018 | 18197 | 9.27E-13 |
| *EN* | | | -0.009 | -3.5 | 0.999683 | 38.4 | 0.999754 | 0.001 | 8386 | 0.999855 |

[1] Difference between the mean correlations of ref with x1 and x2

[2] T-value derived by T test with paired variables

[3] P-value derived by T test with paired variables for x1 > x2

[4] The proportion of cases for which x1 > x2

[5] P-value derived by Binomial test for x1 > x2

[6] Difference between the median correlations of ref with x1 and x2

[7] W-value derived by Wilcoxon matched-paired signed-rank test

[8] P-value derived by Wilcoxon matched-paired signed-rank test for x1 > x2

**TABLE S7**

**Comparison between WCN and $RSA^T$ using sequence variability measures as reference**

| ref. | x1 | x2 | Δ[1] | T[2] | Paired.T[3] | %[4] | Binomial[5] | d[6] | W[7] | Paired.W[8] |
|---|---|---|---|---|---|---|---|---|---|---|
| *CS* | | | 0.049 | 11.9 | 9.91E-26 | 79.2 | 8.65E-19 | 0.057 | 20388 | 2.10E-21 |
| *ET* | | | 0.056 | 12.3 | 4.45E-27 | 79.6 | 2.24E-19 | 0.059 | 20606 | 2.13E-22 |
| *KBSP* | WCN | $RSA^T$ | 0.059 | 12.4 | 2.03E-27 | 81.9 | 1.70E-22 | 0.064 | 20719 | 6.37E-23 |
| *VTSP* | | | 0.060 | 12.6 | 4.56E-28 | 82.9 | 7.78E-24 | 0.063 | 20805 | 2.52E-23 |
| *EN* | | | 0.048 | 10.3 | 1.07E-20 | 77.3 | 1.46E-16 | 0.067 | 19760 | 1.12E-18 |

[1] Difference between the mean correlations of ref with x1 and x2

[2] T-value derived by T test with paired variables

[3] P-value derived by T test with paired variables for x1 > x2

[4] The proportion of cases for which x1 > x2

[5] P-value derived by Binomial test for x1 > x2

[6] Difference between the median correlations of ref with x1 and x2

[7] W-value derived by Wilcoxon matched-paired signed-rank test

[8] P-value derived by Wilcoxon matched-paired signed-rank test for x1 > x2

**FIGURE S1**

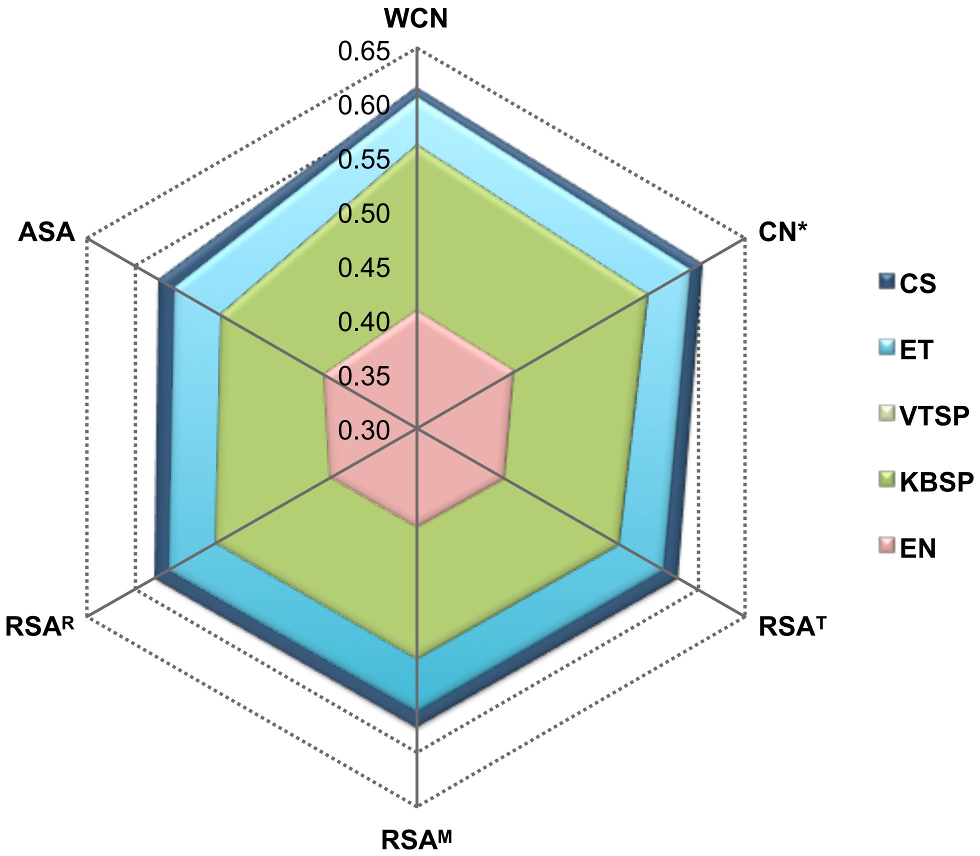


**Figure S1:** Comparison of sequence variability profiles by their average Spearman's correlation coefficients with different structural profiles. The sequence variability scores (listed in the figure legend) are: ConSurf rate of evolution (CS), Evolutionary Trace score (ET), Karlin & Brocchieri Sum-of-Pairs score (KBSP), Valdar & Thornton Sum-of-Pairs score (VTSP), and Entropy (EN). The structural properties (the apices of the hexagon) are: Weighted Contact Number (WCN), Contact Number (CN), Relative Solvent Accessibility (RSA), and Absolute Solvent Accessibility (ASA). The asterisk mark on CN means that the cut-off radius was chosen to maximize each CN-sequence average correlation. The cut-off radii for CS, ET, KBSP, VTSP and EN are 19Å, 19Å, 18Å, 18Å and 20Å, respectively. Superscript letters distinguish RSA profiles obtained using different methods.

**FIGURE S2**

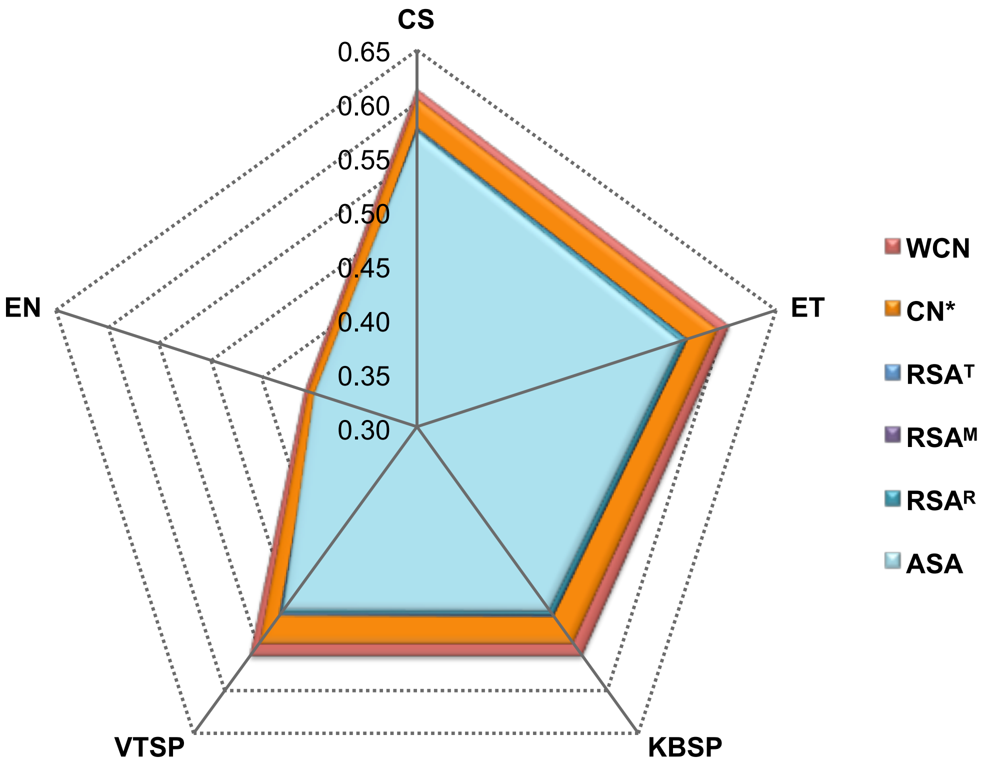


**Figure S2:** Comparison of structural profiles by their average Spearman's correlation coefficients with different sequence variability profiles. The sequence variability measures (the axes of the pentagon) are: ConSurf rate of evolution (CS), Evolutionary Trace score (ET), Karlin & Brocchieri Sum-of-Pairs score (KBSP), Valdar & Thornton Sum-of-Pairs score (VTSP), and Entropy (EN). The structural properties (listed in the figure legend) are: Weighted Contact Number (WCN), Contact Number (CN), Relative Solvent Accessibility (RSA), and Absolute Solvent Accessibility (ASA). The asterisk mark on CN means that the cut-off radius was chosen to maximize each CN-sequence average correlation. The cut-off radii for CS, ET, KBSP, VTSP and EN are 19Å, 19Å, 18Å, 18Å and 20Å, respectively. Superscript letters distinguish RSA profiles obtained using different methods.

**FIGURE S3**

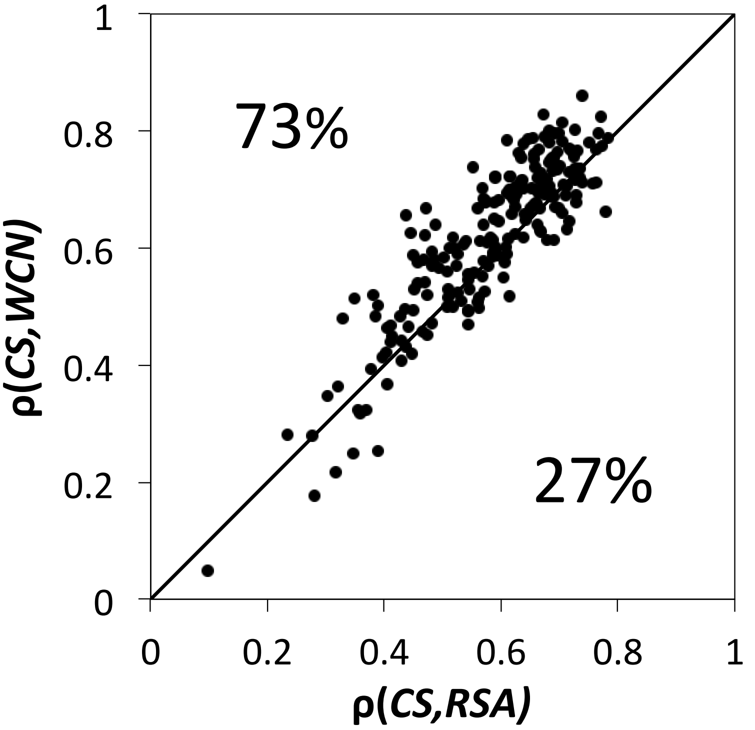


**Figure S3:** Local Packing Density vs. Solvent Accessibility as determinants of site-specific evolutionary rates with Spearman's correlation coefficient. Points above (below) the diagonal are proteins for which WCN ($RSA^T$) correlates better than $RSA^T$ (WCN) with the site-specific rates of amino acid substitution as estimated using the phylogenetic-based approach ConSurf (CS). The percentages of points above and below the diagonals are shown.

**TABLE S8**

**Mean structure-sequence Spearman's correlations**

| **Property** | **Profile** | ***CS*** | ***ET*** | ***KBSP*** | ***VTSP*** | ***EN*** |
|---|---|---|---|---|---|---|
| *LPD* | WCN | 0.614 | 0.605 | 0.561 | 0.561 | 0.408 |
| | CN* | 0.604 | 0.593 | 0.547 | 0.547 | 0.405 |
| *SA* | $RSA^T$ | 0.577 | 0.564 | 0.516 | 0.515 | 0.394 |
| | $RSA^M$ | 0.576 | 0.563 | 0.514 | 0.513 | 0.393 |
| | $RSA^R$ | 0.576 | 0.563 | 0.514 | 0.513 | 0.393 |
| | ASA | 0.573 | 0.558 | 0.509 | 0.508 | 0.399 |

*For each sequence variability profile, the cut-off distance of CN was chosen to maximize the CN-sequence average correlation coefficients. The cut-off radii for CS, ET, KBSP, VTSP and EN are 19Å, 19Å, 18Å, 18Å and 20Å, respectively. Values are the structure-sequence Pearson correlation coefficients averaged over all proteins of the dataset.

**TABLE S9**

**Mean Spearman's correlations between sequence variability measures using RSA as reference**

| ref. | x1 | x2 | Δ[1] | T[2] | Paired.T[3] | %[4] | Binomial[5] | d[6] | W[7] | Paired.W[8] |
|---|---|---|---|---|---|---|---|---|---|---|
| RSA[T] | *CS* | *ET* | 0.013 | 3.1 | 1.26E-03 | 55.6 | 0.058695 | 0.020 | 14061 | 5.43E-03 |
| | *CS* | *KBSP* | 0.061 | 11.3 | 8.20E-24 | 78.2 | 1.19E-17 | 0.070 | 20326 | 3.98E-21 |
| | *CS* | *VTSP* | 0.062 | 11.3 | 5.87E-24 | 77.8 | 4.22E-17 | 0.072 | 20340 | 3.45E-21 |
| | *CS* | *EN* | 0.183 | 18.4 | 2.04E-46 | 95.8 | 2.36E-50 | 0.186 | 23297 | 1.18E-36 |
| | *ET* | *KBSP* | 0.048 | 8.9 | 9.43E-17 | 74.5 | 1.49E-13 | 0.051 | 19372 | 4.30E-17 |
| | *ET* | *VTSP* | 0.049 | 9.1 | 2.61E-17 | 75.0 | 4.98E-14 | 0.052 | 19454 | 2.02E-17 |
| | *ET* | *EN* | 0.170 | 17.3 | 8.89E-43 | 92.6 | 6.26E-42 | 0.166 | 23150 | 8.86E-36 |
| | *KBSP* | *VTSP* | 0.001 | 2.6 | 5.11E-03 | 51.4 | 0.366894 | 0.001 | 12700 | 0.142913 |
| | *KBSP* | *EN* | 0.122 | 12.0 | 3.85E-26 | 83.3 | 1.59E-24 | 0.116 | 21793 | 3.13E-28 |
| | *VTSP* | *EN* | 0.121 | 11.9 | 1.42E-25 | 82.9 | 7.78E-24 | 0.114 | 21670 | 1.36E-27 |

[1] Difference between the mean correlations of ref with x1 and x2
[2] T-value derived by T test with paired variables
[3] P-value derived by T test with paired variables for x1 > x2
[4] The proportion of cases for which x1 > x2
[5] P-value derived by Binomial test for x1 > x2
[6] Difference between the median correlations of ref with x1 and x2
[7] W-value derived by Wilcoxon matched-paired signed-rank test
[8] P-value derived by Wilcoxon matched-paired signed-rank test for x1 > x2

**Table S10**

**Mean Spearman's correlations between sequence variability measures using WCN as reference**

| ref. | x1 | x2 | Δ[1] | T[2] | Paired.T[3] | %[4] | Binomial[5] | d[6] | W[7] | Paired.W[8] |
|---|---|---|---|---|---|---|---|---|---|---|
| WCN | *CS* | *ET* | 0.009 | 1.9 | 0.026554 | 46.3 | 0.876334 | 0.015 | 12376 | 0.237307 |
| | *CS* | *KBSP* | 0.053 | 8.4 | 2.25E-15 | 70.8 | 4.01E-10 | 0.067 | 18553 | 5.34E-14 |
| | *CS* | *VTSP* | 0.054 | 8.4 | 2.31E-15 | 71.8 | 6.37E-11 | 0.068 | 18538 | 6.05E-14 |
| | *CS* | *EN* | 0.206 | 18.1 | 2.17E-45 | 95.8 | 2.36E-50 | 0.207 | 23238 | 2.66E-36 |
| | *ET* | *KBSP* | 0.044 | 6.6 | 1.60E-10 | 67.1 | 2.66E-07 | 0.052 | 17814 | 1.70E-11 |
| | *ET* | *VTSP* | 0.045 | 6.7 | 1.08E-10 | 66.7 | 5.46E-07 | 0.053 | 17806 | 1.80E-11 |
| | *ET* | *EN* | 0.197 | 17.0 | 4.24E-42 | 94.4 | 1.59E-46 | 0.192 | 23013 | 5.65E-35 |
| | *KBSP* | *VTSP* | 0.000 | 1.0 | 0.152483 | 44.9 | 0.941305 | 0.002 | 10906 | 0.811529 |
| | *KBSP* | *EN* | 0.153 | 12.7 | 4.17E-28 | 86.6 | 8.86E-30 | 0.140 | 22056 | 1.28E-29 |
| | *VTSP* | *EN* | 0.152 | 12.6 | 7.14E-28 | 86.1 | 5.56E-29 | 0.138 | 21993 | 2.77E-29 |

[1] Difference between the mean correlations of ref with x1 and x2

[2] T-value derived by T test with paired variables

[3] P-value derived by T test with paired variables for x1 > x2

[4] The proportion of cases for which x1 > x2

[5] P-value derived by Binomial test for x1 > x2

[6] Difference between the median correlations of ref with x1 and x2

[7] W-value derived by Wilcoxon matched-paired signed-rank test

[8] P-value derived by Wilcoxon matched-paired signed-rank test for x1 > x2

**TABLE S11**

**Comparison mean Spearman's correlations between SA profiles using sequence variability measures as reference**

| ref. | x1 | x2 | Δ[1] | T[2] | Paired.T[3] | %[4] | Binomial[5] | d[6] | W[7] | Paired.W[8] |
|---|---|---|---|---|---|---|---|---|---|---|
| *CS* | $RSA^{T}$ | $RSA^{M}$ | 0.001 | 3.1 | 9.63E-04 | 64.4 | 1.48E-05 | 0.004 | 15128 | 1.05E-04 |
| | $RSA^{T}$ | $RSA^{R}$ | 0.001 | 3.1 | 9.65E-04 | 60.6 | 1.06E-03 | 0.005 | 14953 | 2.18E-04 |
| | $RSA^{T}$ | ASA | 0.004 | 2.4 | 9.41E-03 | 59.3 | 3.91E-03 | 0.001 | 14075 | 5.20E-03 |
| | $RSA^{M}$ | $RSA^{R}$ | 0.000 | 0.0 | 0.514708 | 46.3 | 0.876334 | 0.001 | 11062 | 0.762357 |
| | $RSA^{M}$ | ASA | 0.003 | 1.6 | 0.054505 | 56.9 | 0.024111 | -0.003 | 13390 | 0.034558 |
| | $RSA^{R}$ | ASA | 0.003 | 1.6 | 0.052425 | 55.6 | 0.058695 | -0.003 | 13412 | 0.032768 |
| *ET* | $RSA^{T}$ | $RSA^{M}$ | 0.001 | 1.7 | 0.047994 | 54.2 | 0.123666 | 0.002 | 13329 | 0.039945 |
| | $RSA^{T}$ | $RSA^{R}$ | 0.001 | 2.0 | 0.021647 | 55.1 | 0.076439 | 0.003 | 13654 | 0.017657 |
| | $RSA^{T}$ | ASA | 0.006 | 3.8 | 1.02E-04 | 59.3 | 3.91E-03 | 0.002 | 15266 | 5.72E-05 |
| | $RSA^{M}$ | $RSA^{R}$ | 0.000 | 0.9 | 0.175332 | 49.5 | 0.580842 | 0.001 | 12219 | 0.293130 |
| | $RSA^{M}$ | ASA | 0.005 | 3.2 | 7.10E-04 | 58.8 | 5.82E-03 | 0.000 | 14910 | 2.60E-04 |
| | $RSA^{R}$ | ASA | 0.005 | 3.2 | 8.96E-04 | 57.9 | 0.012260 | 0.000 | 14799 | 4.04E-04 |
| *KBSP* | $RSA^{T}$ | $RSA^{M}$ | 0.002 | 7.7 | 2.96E-13 | 72.7 | 9.26E-12 | 0.002 | 18540 | 5.95E-14 |
| | $RSA^{T}$ | $RSA^{R}$ | 0.002 | 7.1 | 9.99E-12 | 69.9 | 2.31E-09 | 0.002 | 17854 | 1.26E-11 |
| | $RSA^{T}$ | ASA | 0.007 | 4.3 | 1.50E-05 | 65.7 | 2.17E-06 | 0.005 | 15988 | 1.72E-06 |
| | $RSA^{M}$ | $RSA^{R}$ | 0.000 | -0.4 | 0.663093 | 46.3 | 0.876334 | 0.000 | 10947 | 0.799254 |
| | $RSA^{M}$ | ASA | 0.005 | 2.9 | 1.95E-03 | 62.0 | 2.46E-04 | 0.004 | 14806 | 3.93E-04 |
| | $RSA^{R}$ | ASA | 0.005 | 3.0 | 1.62E-03 | 62.0 | 2.46E-04 | 0.004 | 14775 | 4.44E-04 |
| *VTSP* | $RSA^{T}$ | $RSA^{M}$ | 0.002 | 7.8 | 1.43E-13 | 73.6 | 1.23E-12 | 0.002 | 18619 | 3.10E-14 |
| | $RSA^{T}$ | $RSA^{R}$ | 0.002 | 7.2 | 4.66E-12 | 69.9 | 2.31E-09 | 0.002 | 17995 | 4.39E-12 |
| | $RSA^{T}$ | ASA | 0.007 | 4.2 | 1.75E-05 | 65.7 | 2.17E-06 | 0.000 | 15936 | 2.26E-06 |
| | $RSA^{M}$ | $RSA^{R}$ | 0.000 | -0.4 | 0.644904 | 46.3 | 0.876334 | 0.000 | 10981 | 0.788720 |
| | $RSA^{M}$ | ASA | 0.005 | 2.9 | 2.23E-03 | 62.5 | 1.45E-04 | -0.002 | 14769 | 4.55E-04 |
| | $RSA^{R}$ | ASA | 0.005 | 2.9 | 1.88E-03 | 63.0 | 8.44E-05 | -0.001 | 14741 | 5.07E-04 |
| *EN* | ASA | $RSA^{T}$ | 0.005 | 3.4 | 4.64E-04 | 61.6 | 4.08E-04 | 0.012 | 15048 | 1.47E-04 |
| | ASA | $RSA^{M}$ | 0.006 | 3.5 | 2.42E-04 | 59.7 | 2.58E-03 | 0.011 | 15207 | 7.43E-05 |
| | ASA | $RSA^{R}$ | 0.006 | 3.6 | 1.90E-04 | 61.1 | 6.65E-04 | 0.010 | 15241 | 6.39E-05 |
| | $RSA^{T}$ | $RSA^{M}$ | 0.001 | 2.2 | 0.016007 | 63.4 | 4.81E-05 | -0.001 | 14384 | 1.87E-03 |
| | $RSA^{T}$ | $RSA^{R}$ | 0.001 | 2.5 | 7.43E-03 | 60.6 | 1.06E-03 | -0.002 | 14173 | 3.80E-03 |
| | $RSA^{M}$ | $RSA^{R}$ | 0.000 | 0.8 | 0.201854 | 51.4 | 0.366894 | -0.001 | 12423 | 0.221808 |

[1] Difference between the mean correlations of ref with x1 and x2
[2] T-value derived by T test with paired variables
[3] P-value derived by T test with paired variables for x1 > x2
[4] The proportion of cases for which x1 > x2
[5] P-value derived by Binomial test for x1 > x2
[6] Difference between the median correlations of ref with x1 and x2
[7] W-value derived by Wilcoxon matched-paired signed-rank test
[8] P-value derived by Wilcoxon matched-paired signed-rank test for x1 > x2

**TABLE S12**

**Mean Spearman correlations between $CN_{cut\text{-}off}$ and sequence profiles**

| Profile | CS | ET | KBSP | VTSP | EN |
|---|---|---|---|---|---|
| $CN_{9}$ | 0.5375 | 0.5271 | 0.4892 | 0.4884 | 0.3651 |
| $CN_{10}$ | 0.5631 | 0.5497 | 0.5104 | 0.5097 | 0.3856 |
| $CN_{11}$ | 0.5685 | 0.5549 | 0.5210 | 0.5207 | 0.3844 |
| $CN_{12}$ | 0.5810 | 0.5677 | 0.5305 | 0.5301 | 0.3931 |
| $CN_{13}$ | 0.5856 | 0.5737 | 0.5360 | 0.5357 | 0.3950 |
| $CN_{14}$ | 0.5905 | 0.5794 | 0.5409 | 0.5405 | 0.3973 |
| $CN_{15}$ | 0.5909 | 0.5802 | 0.5421 | 0.5418 | 0.3962 |
| $CN_{16}$ | 0.5939 | 0.5837 | 0.5444 | 0.5441 | 0.3976 |
| $CN_{17}$ | 0.5980 | 0.5879 | 0.5460 | 0.5457 | 0.4002 |
| $CN_{18}$ | 0.6023 | 0.5922 | 0.5469 | 0.5465 | 0.4033 |
| $CN_{19}$ | 0.6037 | 0.5934 | 0.5457 | 0.5453 | 0.4045 |
| $CN_{20}$ | 0.6035 | 0.5931 | 0.5437 | 0.5432 | 0.4048 |
| $CN_{21}$ | 0.6029 | 0.5925 | 0.5412 | 0.5406 | 0.4039 |
| $CN_{22}$ | 0.6020 | 0.5916 | 0.5385 | 0.5379 | 0.4025 |
| $CN_{23}$ | 0.5993 | 0.5893 | 0.5351 | 0.5345 | 0.4000 |
| $CN_{24}$ | 0.5963 | 0.5866 | 0.5312 | 0.5306 | 0.3976 |
| $CN_{25}$ | 0.5924 | 0.5832 | 0.5265 | 0.5258 | 0.3939 |
| $CN_{26}$ | 0.5888 | 0.5799 | 0.5219 | 0.5213 | 0.3910 |
| $CN_{27}$ | 0.5837 | 0.5751 | 0.5160 | 0.5153 | 0.3863 |
| $CN_{28}$ | 0.5783 | 0.5703 | 0.5105 | 0.5098 | 0.3822 |
| $CN_{29}$ | 0.5726 | 0.5648 | 0.5040 | 0.5033 | 0.3779 |
| $CN_{30}$ | 0.5665 | 0.5589 | 0.4976 | 0.4968 | 0.3734 |

**TABLE S13**

**Comparison mean Spearman's correlations between LPD profiles using sequence variability measures as reference**

| ref. | x1 | x2 | Δ[1] | T[2] | Paired.T[3] | %[4] | Binomial[5] | d[6] | W[7] | Paired.W[8] |
|---|---|---|---|---|---|---|---|---|---|---|
| *CS* | | | 0.011 | 4.9 | 1.01E-06 | 66.2 | 1.10E-06 | 0.017 | 16517 | 9.04E-08 |
| *ET* | | | 0.012 | 5.2 | 2.04E-07 | 65.3 | 4.19E-06 | 0.024 | 16571 | 6.57E-08 |
| *KBSP* | WCN | CN* | 0.014 | 6.6 | 1.86E-10 | 66.7 | 5.46E-07 | 0.015 | 17386 | 3.57E-10 |
| *VTSP* | | | 0.014 | 6.6 | 2.05E-10 | 67.6 | 1.27E-07 | 0.013 | 17401 | 3.22E-10 |
| *EN* | | | 0.003 | 1.4 | 0.076363 | 53.2 | 0.188224 | 0.007 | 13086 | 0.068498 |

1 Difference between the mean correlations of ref with x1 and x2
2 T-value derived by T test with paired variables
3 P-value derived by T test with paired variables for x1 > x2
4 The proportion of cases for which x1 > x2
5 P-value derived by Binomial test for x1 > x2
6 Difference between the median correlations of ref with x1 and x2
7 W-value derived by Wilcoxon matched-paired signed-rank test
8 P-value derived by Wilcoxon matched-paired signed-rank test for x1 > x2

**TABLE S14**
**Comparison mean Spearman's correlations between WCN and RSA[T] using sequence variability measures as reference**

| ref. | x1 | x2 | Δ[1] | T[2] | Paired.T[3] | %[4] | Binomial[5] | d[6] | W[7] | Paired.W[8] |
|---|---|---|---|---|---|---|---|---|---|---|
| *CS* | | | 0.037 | 8.4 | 3.67E-15 | 72.7 | 9.26E-12 | 0.045 | 18437 | 1.38E-13 |
| *ET* | | | 0.041 | 8.8 | 2.45E-16 | 75.9 | 5.21E-15 | 0.050 | 18860 | 4.05E-15 |
| *KBSP* | WCN | RSA[T] | 0.045 | 9.5 | 1.58E-18 | 75.0 | 4.98E-14 | 0.049 | 19452 | 2.06E-17 |
| *VTSP* | | | 0.046 | 9.7 | 6.42E-19 | 75.9 | 5.21E-15 | 0.048 | 19547 | 8.47E-18 |
| *EN* | | | 0.014 | 3.2 | 7.77E-04 | 56.5 | 0.032972 | 0.024 | 14495 | 1.27E-03 |

[1] Difference between the mean correlations of ref with x1 and x2
[2] T-value derived by T test with paired variables
[3] P-value derived by T test with paired variables for x1 > x2
[4] The proportion of cases for which x1 > x2
[5] P-value derived by Binomial test for x1 > x2
[6] Difference between the median correlations of ref with x1 and x2
[7] W-value derived by Wilcoxon matched-paired signed-rank test
[8] P-value derived by Wilcoxon matched-paired signed-rank test for x1 > x2

**TABLE S15**

**Variance partitioning of Spearman's correlations**

| **Fit** | **Contribution** | **$R^2$** | **%** |
|---|---|---|---|
| $CS \sim WCN+RSA^T$ | Total | 0.420±0.010 | 100 |
| | Common | 0.332±0.009 | 76.43±1.37 |
| | Unique WCN | 0.069±0.004 | 16.85±1.04 |
| | Unique $RSA^T$ | 0.020±0.002 | 6.72±0.84 |

NOTE: Fit is the bivariate linear fit considered, $R^2$ is the explained variance averaged over the dataset of 216 enzymes ± its standard deviation, % is the proportion of explained variance accounted for by the given contribution.
